# Epidrug Repurposing: Discovering New Faces of Old Acquaintances in Cancer Therapy

**DOI:** 10.3389/fonc.2020.605386

**Published:** 2020-11-18

**Authors:** Michel Montalvo-Casimiro, Rodrigo González-Barrios, Marco Antonio Meraz-Rodriguez, Vasti Thamara Juárez-González, Cristian Arriaga-Canon, Luis A. Herrera

**Affiliations:** ^1^ Unidad de Investigación Biomédica en Cáncer, Instituto Nacional de Cancerología-Instituto de Investigaciones Biomédicas, UNAM, Mexico City, Mexico; ^2^ Departamento de Bioquímica, Facultad de Química, Universidad Nacional Autónoma de México, Mexico City, Mexico; ^3^ Instituto Nacional de Medicina Genómica, Mexico City, Mexico

**Keywords:** epidrugs, drug repurposing, cancer therapy, cancer, epigenetic inhibitors, epigenetics

## Abstract

Gene mutations are strongly associated with tumor progression and are well known in cancer development. However, recently discovered epigenetic alterations have shown the potential to greatly influence tumoral response to therapy regimens. Such epigenetic alterations have proven to be dynamic, and thus could be restored. Due to their reversible nature, the promising opportunity to improve chemotherapy response using epigenetic therapy has arisen. Beyond helping to understand the biology of the disease, the use of modern clinical epigenetics is being incorporated into the management of the cancer patient. Potential epidrug candidates can be found through a process known as drug repositioning or repurposing, a promising strategy for the discovery of novel potential targets in already approved drugs. At present, novel epidrug candidates have been identified in preclinical studies and some others are currently being tested in clinical trials, ready to be repositioned. This epidrug repurposing could circumvent the classic paradigm where the main focus is the development of agents with one indication only, while giving patients lower cost therapies and a novel precision medical approach to optimize treatment efficacy and reduce toxicity. This review focuses on the main approved epidrugs, and their druggable targets, that are currently being used in cancer therapy. Also, we highlight the importance of epidrug repurposing by the rediscovery of known chemical entities that may enhance epigenetic therapy in cancer, contributing to the development of precision medicine in oncology.

## Introduction

Since the turn of the century, epigenetics has become an important research area in human diseases study, where genetic mutations have been classically understood as the main cause in the development of human pathologies (1). The term epigenetics involves a wide variety of mechanisms that cells use to regulate the transcription of their DNA without changing its genetic material (2). Whether an epigenetic modification has a facilitating or inhibiting role in the gene expression depends on the chemical nature of the mark that is placed over the chromatin, and the type of modification that is set down on the proximal environment of these genes (3). Thus, epigenetics shapes a regulatory complex that bridges the gap between genetic sequences and actionable mutations. Due to current knowledge about these epigenetic mechanisms, the importance of this regulatory system has become more evident and it has led to the understanding that epigenetic alterations are some of the main mechanisms underlying many human diseases such as cancer, which arises through aberrant genetic and epigenetic alterations, both of which have a key role in malignant transformation, tumor progression and prognosis (4).

Nowadays, it is known that as cancer progresses, there are genetic aberrations that make tumors highly prone to developing resistance to therapies (5). Emerging data on cancer-associated epigenetic alterations have shown that epigenetic modifications leading to drug resistance may be the cue for individual variation in chemotherapy response, having the potential to be reversible using epigenetic therapy (6). The possibility to reprogram the cancer epigenome is becoming a promising target therapy for both, treatment development and reversibility of drug resistance. Which focuses on the development of pharmacological compounds that can reprogram the epigenetic landscape to enhance chemotherapy response (7).

For a few years, the design of therapeutic strategies has been a growing field of query for single-target epigenetic drugs (epidrugs); however, the traditional epidrug discovery pathway is time-consuming and expensive (8, 9). Hence, a promising strategy for epidrug development is based on tracing novel potential epi-targets in previously approved drugs through a process called drug repositioning or repurposing (10, 11). Epidrug repurposing allows exploring a wide diversity of molecular combinations in multifactorial diseases such as cancer, where combinational epigenetic therapies are likely to be more effective than monotherapy to overcome chemotherapy resistance (9). This review focuses on the emerging area of ​​epidrug repurposing, highlighting strategies to enhance cancer therapy. To further understand this, we will discuss the main mechanisms and elements involved in epigenetic alterations in cancer and its relevance in cancer therapy response.

### Background in Epigenetics

Epigenetics is the term coined by Conrad Hal Waddington seventy-six years ago, to refer to the molecular mechanisms that may exert their influence on gene expression that do not involve alterations in its gene code. Through these, an organism can develop and adapt its phenotype to environmental changes (12). Over time, many definitions of Epigenetics have arisen (13); however, we can understand epigenetics as reversible chemical modifications of DNA and histone proteins (epimarks) that regulate specific functions in chromatin remodeling without altering the DNA sequence (14). Epimarks are associated with the transcription and function of a gene, that may change the cellular phenotype or its functional patterns in response to a particular context, across different developmental stages, cellular differentiation, or maintenance of tissue-specific cell lineages (15).

**Figure 1:**
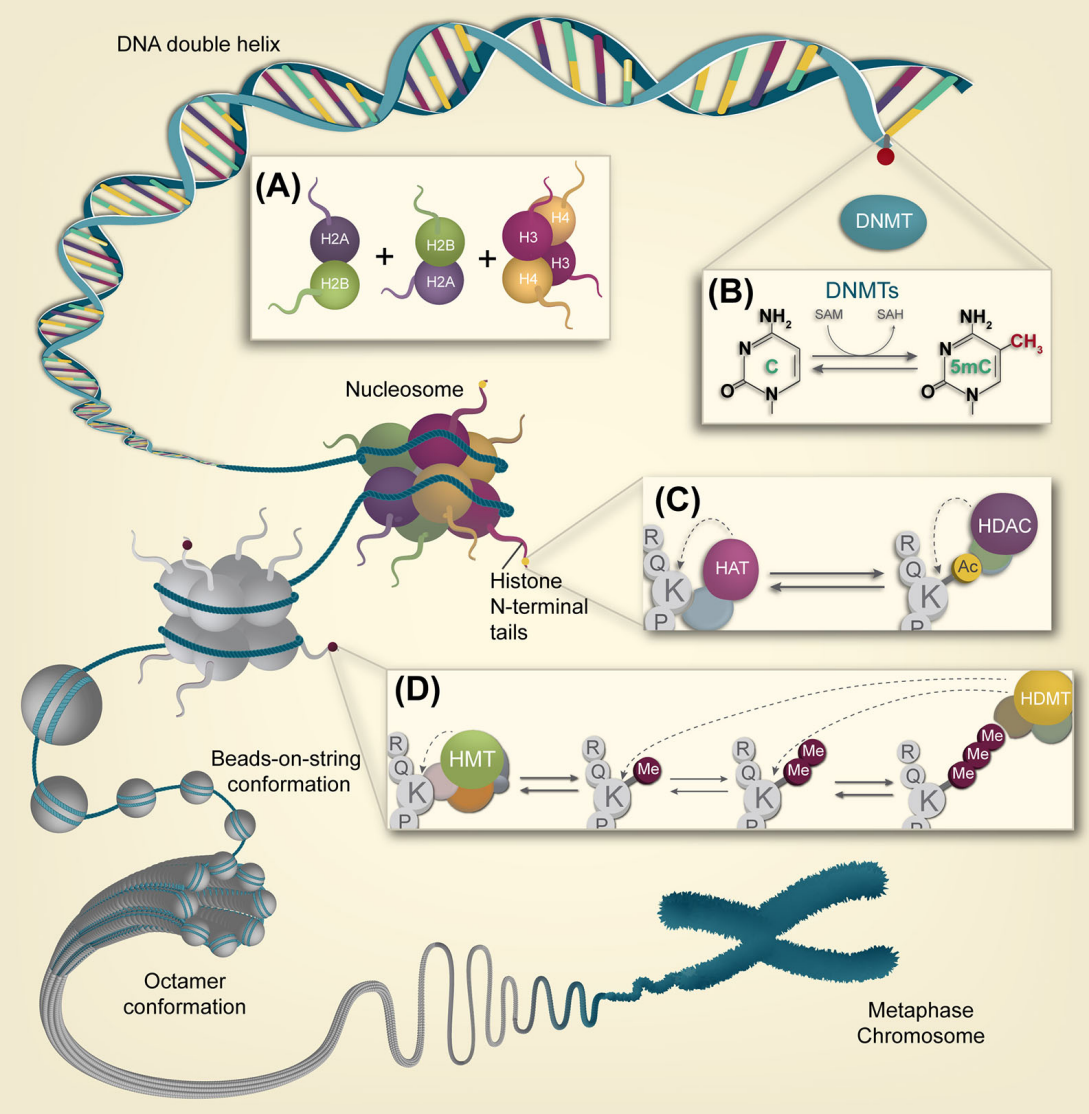
Overview of the epigenetic landscape. Different compaction levels of chromatin are depicted, from naked DNA to the metaphasic chromosome. **(A)** Two dimers of H2A-H2B and a tetramer of H3-H4 histones are required for nucleosome assembly, the chromatin’s basic packaging unit **(B)** DNA methylation is a process carried out by DMNTs in CpG dinucleotides, particularly on CpG islands. This dynamic epigenetic mark can be reversed by enzymatic conversion. **(C)** Histone acetylation is performed on lysine residues by HAT enzyme complexes. In contrast, histone lysine deacetylation is carried out by HDACs enzyme complexes. **(D)** Histone lysine methylation is carried out by HMT complexes. Lysines can be processively methylated from mono to di and trimethylation.

At the molecular level, epigenetic machinery is composed mainly of three interconnected components working synergistically in the chromatin organization levels, which include DNA methylation, histone post-translational modifications, and regulatory non-coding RNAs (ncRNAs) (14, 16). In the nucleus, chromatin can exist in two physical and functional states: heterochromatin (condensed chromatin), which is associated with transcriptional repression; and euchromatin (relaxed chromatin), associated with transcriptional activation (17) (**Figure 1**). The organizational states of the chromatin are highly regulated by epigenetic mechanisms involving nucleosome, which is the basic packaging unit of chromatin, composed by an octamer of histone proteins (two dimers of H2A-H2B and a tetramer of H3-H4 histones) (**Figure 1A**), that constitutes a compact structure with 147 base pairs of DNA turned almost twice around it (17, 18). N-terminal tails of histone proteins can acquire post-translational modifications through multiple mechanisms including phosphorylation, ubiquitination, methylation/demethylation, and acetylation, the latter being the most studied. Histone and direct DNA modifications constitute “the epigenetic code”: an interplay between epigenetic factors and positive and negative feedback mechanisms that regulate it (18). Therefore, understanding the main mechanisms in the field of epigenetic research and their role in disease development is essential in its application in cancer therapy.

### DNA Methylation

Methylation on DNA’s cytosine is the most broadly studied epigenetic modification in humans. It encompasses a reaction defined as “the covalent transfer of a methyl group to the C-5 position of a cytosine ring of DNA” (15, 19). Generally, in mammals, DNA methylation occurs predominantly—but not exclusively—in the context of genomic regions called CpG islands, which are formed by clusters of CpG dinucleotides, and it’s catalyzed by a group of enzymes called DNA methyltransferases (DNMTs). These enzymes transfer a methyl group from the donor molecule S-adenosylmethionine (SAM) to the fifth carbon of a cytosine residue to form 5-methylcytosine (5mC) (18, 19) (**Figure 1B**). This covalent modification is able to inhibit DNA transcription; either through the steric hindrance imposed by the methyl group which prevents transcription factors from binding DNA (18–20), or by the recruitment of proteins with methyl-CpG-binding domains (MBD). These proteins also contain domains able to recruit histone-modifying and chromatin-remodeling complexes to the methylated sites, forming repressor complexes that enhance the silencing state on that chromatin region (21). Three different DNMTs generate and maintain methylation patterns. DNMT1 is the methyltransferase enzyme specialized in the maintenance of previously placed methylation patterns, and DNMT3a & DNMT3b are instead involved in the establishment of *de novo* methylation patterns over DNA (18, 22, 23).

DNA methylation patterns occur in different regions of the genome. Alterations in these patterns lead to diseases (18). For instance, gene promoters which are mainly embedded in CpG islands (70%) are normally unmethylated, thus allowing transcription. Aberrant hypermethylation patterns of these gene regulatory elements lead to transcriptional inactivation and are tumor-type specific as well as a common hallmark of cancer (9). Alternatively, during diseases, other alterations occur, like the demethylation of the gene body. Such alteration allows transcription to be initiated at several incorrect sites. In consequence, DNA hypomethylation at specific regions can activate the aberrant expression of genes, some of which could behave as proto-oncogenes (18). Finally, as aforementioned, alterations of hypermethylated patterns in repetitive sequences can promote the activation of transposable elements and chromosomal instability, both phenomena being also correlated with carcinogenesis and metastasis (6).

However, the reactions that lead to altered patterns of DNA methylation can potentially be reversible and restored through DNMT inhibitors (DNMTi: see below) that contain nucleoside derivatives and non-nucleoside analogs, some of them have been highly researched and shown promise in cancer therapies (24).

### Histone Post-Translational Modifications

Another axis of the epigenetic machinery, closely associated with DNA methylation, are the covalent post-translational modifications of nucleosomal histones. Through the addition of chemical groups at specific sites within the amino- or carboxy-terminus of each histone, different functional consequences influencing chromosome structure can be elicited. Chromatin is functionally divided into actively transcribed euchromatin and transcriptionally inactive heterochromatin, which finally regulates the accessibility to genomic DNA and has a role in the control of gene expression (18, 25). The principal histone proteins modifications include methylation, acetylation, phosphorylation, ubiquitylation, sumoylation, and ribosylation, from which methylation and acetylation are the most common and characterized, and generally occur in the proximity of promoter and enhancer genomic regions (26). Each histone residue can undergo one or more modifications, which have different effects depending on which residue is modified, giving rise to crosstalk between the different marks, constituting “the histone code” altogether (18).

Multiple enzymes catalyze histone post-translational modifications with specific catalytic activity based on each histone tail’s amino acids that can act as their substrates. Most of these modifications are reversible. There are specialized enzymes that can remove each type of covalent modification. Histone acetyltransferases (HATs) and deacetylases (HDACs) control acetylation, as well as histone methyltransferases (HMTs) and demethylases (HDMs) coordinate histone methylation. Acetylation and deacetylation of histones are among the most studied reversible, followed by methylation and demethylation of histone lysines (17, 27).

Due to the importance of histone epimarks in gene regulation and cellular function, aberrant histone post-translational modifications may change gene expression patterns and cause human pathologies (6). Thus, it is of great importance to understand the reversible nature of these marks as an advantageous alternative for the treatment of diseases where epigenome deregulation is one of the hallmarks. 

### Histone Acetylation and Deacetylation

Histone acetylation has a key role in many biological processes (cell cycle regulation, alternative splicing, nuclear transport, among others) (28). It can promote relaxed states of the chromatin (euchromatin) and favor gene transcription, while deacetylation exerts the opposite effect, generating heterochromatin domains that can inhibit transcription (2). Two families of enzymes with reverse functions control the feedback regulation between acetylation/deacetylation of histones: histones acetyltransferases (HATs or KATs) and histones deacetylases (HDACs) (2). HATs catalyze the transfer of acetyl groups to lysine-amino-terminal residues using acetyl-CoA as a donor; this reaction neutralizes the positive charge of the Lys (17, 29) (**Figure 1C**). As a result, the interaction between the histone and the DNA is weakened, forming an opening domain in chromatin, leading to exposure of DNA sequences and their transcription (2, 28). HATs are divided in three families based on their catalytic domain’s functional and structural identity, which bears the acetyltransferase activity for the recognition of acetyl-lysine residues (17). Several HATs associate with other protein complexes and subunits to selectively modify the different histones; however, p300/CBP is probably the most extensively studied HAT, since it is capable of acetylating all four histones along with many other coactivator or corepressor transcriptional complexes (30).

In contrast, HDACs remove acetyl groups from lysine residues through different reactions that reestablish the positive charges on histone tails, increasing its interaction with DNA and stabilizing the chromatin in place (2, 28) (**Figure 1C**). The histone deacetylase family includes 18 members (31), divided into two groups based on their enzymatic activity: Zn^2+^-dependent enzymes, which include classes I, II, and IV HDACs, exert their function through hydrolytic catalysis; and NAD+ cofactor-dependent enzymes, that include class III sirtuins (SIRTs), with a catalytic mechanism of nucleophilic substitution for histone deacetylation (28).

Both HATs and HDACs play a key role in the maintenance and regulation of chromatin accessibility, leading gene expression regulation, among other mechanisms. Histone acetylation global imbalance is one of the prominent alterations in the diseased state and a hallmark of many tumor types, where HDACs have been found overexpressed (32) or mutated (33). Additionally, abnormal genomic events such as translocations, mutations, or deletions in HAT- and acetylation readers-related genes may occur during cancer development (18). As a result, aberrant acetylation-related proteins contribute to the progression of the disease. For instance, germline mutations and overexpression of HDACs have been observed in various cancers, resulting in a global loss of histone acetylation and the consequent silencing of tumor suppressor genes (34). Also, it has been observed that reduced lysine 16 acetylation (H4K16ac), as well as the loss of acetylation of histone 3 (H3ac) are also hallmarks of human cancer (35, 36). Furthermore, HATs and HDACs are targeted to transcriptionally-active genes by phosphorylated RNA polymerase II through the recruitment of effector proteins with specialized reader domains (18), suggesting that the mechanistic switch between acetylation/deacetylation can be manipulated and restored by specific drugs inhibiting key enzymes by targeting their catalytic reaction (HATi and HDACi; see below).

### Histone Methylation and Demethylation

Histone methylation occurs on arginine (R) and lysine (K) residues, and it is catalyzed by HMTs (or KMTs and RMTs) that use S-adenosyl-l-methionine (SAM) as a methyl donor group (**Figure 1C**). Lysine methyltransferases are divided into two broad groups based on the presence or the absence of a SET domain (Su(var)3-9, Enhancer-of-zeste, and Trithorax): SET-domain containing methyltransferase family and DOT1-domain lysine N-methyltransferase (37, 38).

KMTs can transfer three methyl groups onto lysine residues, prompting mono, di, and, trimethylation (me1, me2 and, me3 respectively) (17) (**Figure 1D**). The association of an active or repressive transcriptional state depends on the number of methyl groups and in the position of the lysine residue in the histone amino acid sequence. A repressed chromatin state (heterochromatin, constitutive, or facultative), correlates with methylation of H3K9me2,3, H3K27me2,3, and H4K20me3, while methylation of H3K4me2,3, H3K9me1, H3K27me1, H3K20me1, and H3K36me1 are associated with transcriptionally active chromatin (euchromatin) (17, 39). Besides, histone methylation also has an important role in DNA repair, DNA replication, alternative splicing, and chromosome condensation (18). Histone demethylases HDMs (or KDMs) can revert these modifications (**Figure 1D**), divided into two different families with distinct enzymatic mechanisms: KDM1A/LSD1 amine oxidase family, dependent on flavin adenine dinucleotide (FAD) as a cofactor; and the KDM2A/B dioxygenase family, which contain a Jumonji C (JmjC) domain and are iron Fe (II) and α-ketoglutarate-dependent to accomplish histone demethylation through methyl groups oxidation (40). The readers of methylated lysine residues consist of various proteins with specialized domains that can recognize these modifications (17).

Besides the global loss of acetylation and DNA hypomethylation, the deregulation of histone methylation/demethylation can lead to chromosome instability (18). It has been suggested that the aberrant expression of both histone methyltransferases and demethylases genes is the main cause of an altered distribution of histone methylation marks. Deregulation of histone methylation patterns can become a driver for mutations in many types of tumors (15). For instance, cancer cells have a global loss of activation marks, such as H4K20me3; along with a gain of methylation in repressive marks, such as H3K9me and H3K27me, as well as the monomethylation of H3K4me (35, 36) which are associated with DNA hypermethylation of silenced genes. The basal patterns of histone methylation are essential for establishing a permissive euchromatic state, allowing the expression of tumor suppressor genes. Therefore, its alteration results in the repression of some of these genes and oncogene aberrant expression (18, 35). Instead, instability of the methylation/demethylation mechanistic switch can promote proliferation and neoplastic transformation in several cancer types (41–43).

### Epigenetic Alterations in Cancer and Cancer Therapy

**Figure 2:**
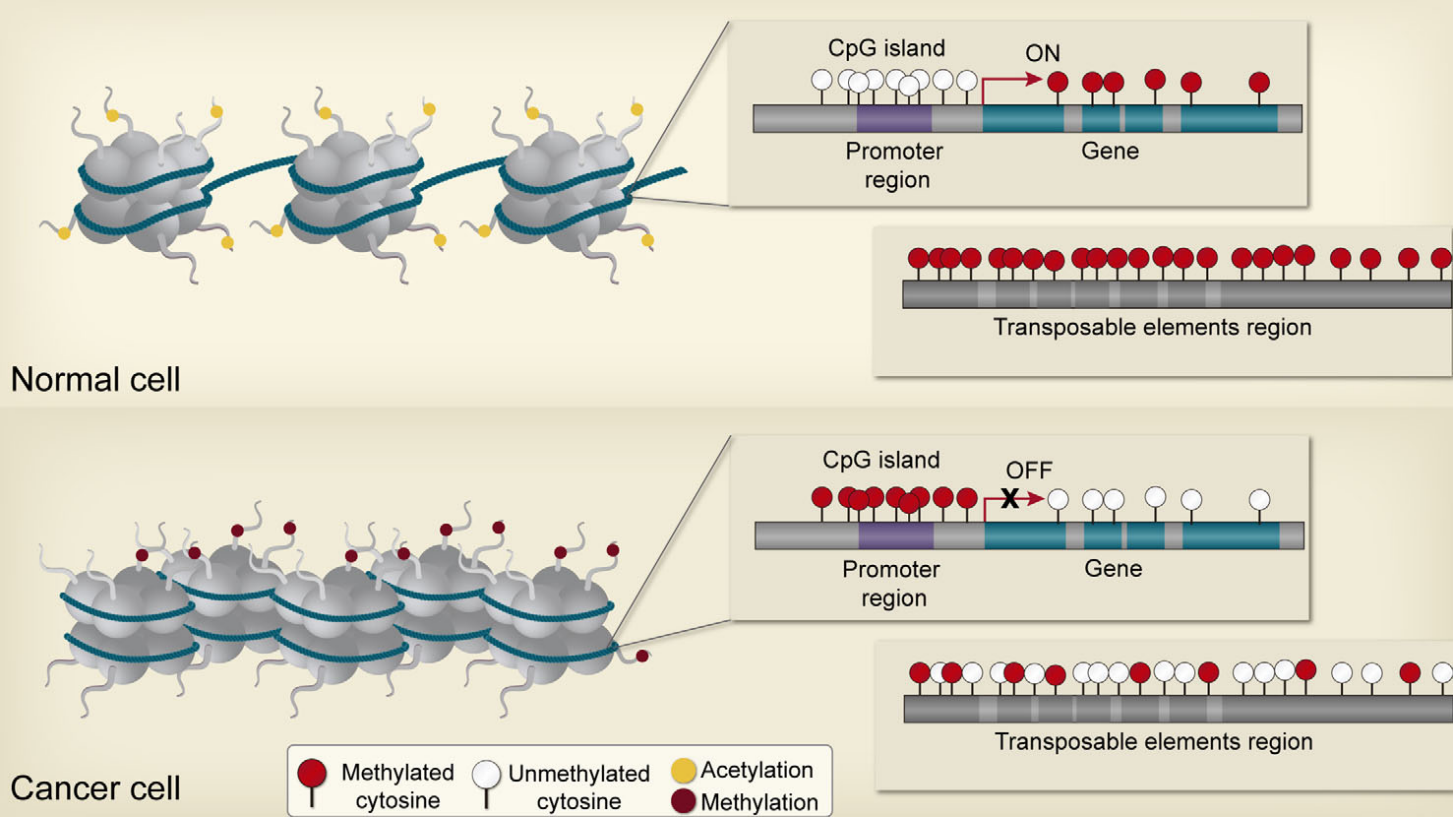
Epigenetic alterations in cancer cells. In non-neoplasic cells, CpG islands of tumor suppressor gene promoters are generally unmethylated and acetylated, resulting in transcriptional activation and expression. In contrast, non-coding regions and repetitive elements are hypermethylated, ensuring chromosome stability. Gene bodies are normally methylated, enhancing transcription. Neoplasic cells are characterized by global hypomethylation and local CpG island hypermethylation, especially at tumor suppressor gene promoters, resulting in aberrant transcription and genomic instability.

As mentioned before, the cancer epigenome is characterized by global changes in DNA methylation, disruptions in histone posttranslational modification patterns, and alterations of normal chromatin-modifying enzymes expression (18, 36) (**Figure 2**) [see review (44)]. Accordingly, these changes can promote the disruption of cellular homeostasis in precancerous cells through the deregulation of genes implicated in cancer initiation and progression (4); for instance, those genes associated with apoptosis resistance, proliferation, invasive potential, and genomic instability, as well as genes correlated to therapeutic response (45, 46). Thus, the relationship between genetic disruptions and epigenetic abnormalities are mutually beneficial in order to drive cancer development and could be playing a key role in individual differences displayed by patients in the way they respond to therapies in both toxicity or treatment efficacy (15, 46, 47). Multiple studies demonstrate that reversing epigenetic patterns through *de novo* epidrugs and epidrug repurposing can resensitize cancer cells to chemotherapy (48–50).

### Principles of Epigenetic Therapy

Increasing understanding of epigenetic mechanisms and their importance in disease has led to the development of therapeutic interventions targeting epigenetic modulatory mechanisms. Due to the chemical reversibility nature of DNA methylation and histone posttranslational modifications, epigenetic proteins can be druggable targets by means of small-enzymatic inhibitors that aim for the restoration of the aberrant epigenetic machinery and hold the potential for reverting epigenetic signatures in cancer (14).

Epigenetic drugs (epidrugs) are chemical agents that modify the structure of DNA and chromatin, facilitating disruption of transcriptional and post-transcription changes, primarily by controlling the enzymes required for their establishment and maintenance, reactivating the tumor suppressor and DNA repair genes that are epigenetically silenced (51). Lately, epigenetic therapy has taken relevance in the field of oncology, where epidrugs have been successfully used in treatment, mostly in combination with standard chemotherapy (52).

Epidrugs (with one-target, as well as repurposed epidrugs; see below) that are designed based on these principles can exert direct cytotoxic effects over malignant cells (14, 46), function as sensitizers in complementary therapies (53, 54), or can be used to overcome epigenetically-acquired drug resistance against the limits of chemotherapy efficacy, as there are the dynamic associations between epigenetic pattern changes and resistance to therapeutic regimes for cancer (50, 52, 55). New epidrugs compounds are continually being tested for cytotoxicity, pharmacological parameters, and a better understanding of their mode of action; in both preclinical research (*in vitro* and *in vivo*) as well as in clinical trials. Epigenetics therapy is enhanced by a combination of laboratory and clinical data. The US Food and Drug Administration (FDA) has approved many epigenetic treatments and used them for treating cancer (6).

### Epidrug Generations

Historically, molecules designed to inhibit the catalytic function of epigenetic factors have not only resulted in the reduction of the targeted enzymatic activity but also the appearance of indirect modifications of the transcription of large gene sets (56). Several epigenetic protein families have similar cofactors and co-substrates, similar epidrugs could target several epigenetic protein families. Some compounds can inhibit the functionality of a whole family of epigenetic proteins (**Table 1**).

**Table 1:** Current inhibition assays performed for different epigenetic factors.

Type of inhibitor		Epigenetic Factor	Acronym	CHEMBL ID	Inhibitor molecules
**DNMTi**		DNA (cytosine-5)-methyltransferase 1	DNMT1	CHEMBL1993	841
		DNA (cytosine-5)-methyltransferase 3A	DNMT3A	CHEMBL1992	258
		DNA (cytosine-5)-methyltransferase 3B	DNMT3B	CHEMBL6095	80
**HDACi**	**HDACi (Zn dependent)**	Histone deacetylase 1	HDAC1	CHEMBL325	6434
		Histone deacetylase 6	HDAC6	CHEMBL1865	4701
		Histone deacetylase 8	HDAC8	CHEMBL3192	2420
		Histone deacetylase 3	HDAC3	CHEMBL1829	2043
		Histone deacetylase 2	HDAC2	CHEMBL1937	2003
		Histone deacetylase 4	HDAC4	CHEMBL3524	1279
		Histone deacetylase 7	HDAC7	CHEMBL2716	521
		Histone deacetylase 11	HDAC11	CHEMBL3310	503
		Histone deacetylase 5	HDAC5	CHEMBL2563	460
		Histone deacetylase 10	HDAC10	CHEMBL5103	419
		Histone deacetylase 9	HDAC9	CHEMBL4145	348
	**SIRTi (NAD+ dependent)**	NAD-dependent deacetylase sirtuin 1	SIRT 1	CHEMBL4506	2073
		NAD-dependent deacetylase sirtuin 2	SIRT 2	CHEMBL4462	2839
		NAD-dependent deacetylase sirtuin 3	SIRT 3	CHEMBL4461	634
		NAD-dependent deacetylase sirtuin 5	SIRT 5	CHEMBL2163183	250
		NAD-dependent deacetylase sirtuin 6	SIRT 6	CHEMBL2163182	221
		NAD-dependent deacetylase sirtuin 7	SIRT 7	CHEMBL2163184	10
**HMTi**	**KMTi**	Histone-lysine N-methyltransferase, H3 lysine-9 specific 5	KMT1D	CHEMBL6031	238
		Histone-lysine N-methyltransferase, H3 lysine-9 specific 3	G9A	CHEMBL6032	92523
		Histone-lysine N-methyltransferase MLL	MLL1	CHEMBL1293299	17219
		Histone-lysine N-methyltransferase EZH2	EZH2	CHEMBL2189110	1243
		Histone-lysine N-methyltransferase, H3 lysine-79 specific	DOT1L	CHEMBL1795117	344
		Histone-lysine N-methyltransferase SETD7	SETD7	CHEMBL5523	204
		Histone-lysine N-lysine methyltransferase SETD8	SETD8	CHEMBL1795176	98
		Histone-lysine N-lysine methyltransferase SMYD2	SMYD2	CHEMBL2169716	84
		Histone-lysine N-methyltransferase SMYD3	SMYD3	CHEMBL2321643	54
		Histone-lysine N-methyltransferase SUV39H1	SMYD2	CHEMBL2169716	84
		Histone-lysine N-methyltransferase EZH1	EZH1	CHEMBL2189116	32
		Histone-lysine N-methyltransferase SUV39H2	SUV39H2	CHEMBL1795177	21
		Histone-lysine N-methyltransferase NSD2	NSD2	CHEMBL3108645	20
		Histone-lysine N-methyltransferase SETDB1	SETDB1	CHEMBL2321646	14
		Histone-lysine N-methyltransferase SUV420H2	SUV420H2	CHEMBL2321644	12
		Histone-lysine N-methyltransferase SETD2	SETD2	CHEMBL3108647	11
		Histone-lysine N-methyltransferase, H3 lysine-36 and H4 lysine-20 specific	NSD1	CHEMBL3588738	10
		Histone-lysine N-methyltransferase PRDM9	PRDM9	CHEMBL3588737	10
		Histone-lysine N-methyltransferase SUV420H1	SUV420H1	CHEMBL2321645	9
		Histone-lysine N-methyltransferase MLL3	MLL3	CHEMBL2189113	7
		Histone-lysine N-methyltransferase NSD3	NSD3	CHEMBL3108646	7
		Histone-lysine N-methyltransferase ASH1L	ASH1L	CHEMBL3588739	6
		Histone-lysine N-methyltransferase SETMAR	SETMAR	CHEMBL2189111	3
		Histone-lysine N-methyltransferase MLL2	MLL2	CHEMBL2189114	2
		Histone-lysine N-methyltransferase MLL4	MLL4	CHEMBL2189112	2
		Histone-lysine N-methyltransferase SETD1B	SET1B	CHEMBL4105837	1
		Histone-lysine N-methyltransferase SETD1A	SETD1A	CHEMBL4105954	1
	**RMTi**	Histone-arginine methyltransferase CARM1	CARM1	CHEMBL5406	201
		Protein-arginine N-methyltransferase 1	PRMT1	CHEMBL5524	528
		Protein arginine N-methyltransferase 6	PRMT6	CHEMBL1275221	139
		Protein arginine N-methyltransferase 3	PRMT3	CHEMBL5891	138
		Protein arginine N-methyltransferase 5	PRMT5	CHEMBL1795116	91
		Protein arginine N-methyltransferase 7	PRMT7	CHEMBL3562175	25
**HDMi**	**JmjC**	Probable JmjC domain-containing histone demethylation protein 2C	JHDM2C	CHEMBL3792271	1
		Histone lysine demethylase PHF8	PHF8	CHEMBL1938212	136
		Lysine-specific demethylase 2A	KDM2A	CHEMBL1938210	128
		Lysine-specific demethylase 2B	KDM2B	CHEMBL3779760	333
		Lysine-specific demethylase 3A	KDM3A	CHEMBL1938209	87
		Lysine-specific demethylase 3B	KDM3B	CHEMBL3784906	9
		Lysine-specific demethylase 4A	KDM4A	CHEMBL5896	51948
		Lysine-specific demethylase 4B	KDM4B	CHEMBL3313832	73
		Lysine-specific demethylase 4C	KDM4C	CHEMBL6175	878
		Lysine-specific demethylase 4D	KDM4D	CHEMBL6138	53
		Lysine-specific demethylase 4D-like	KDM4E	CHEMBL1293226	110
		Lysine-specific demethylase 5A	KDM5A	CHEMBL2424504	621
		Lysine-specific demethylase 5B	KDM5B	CHEMBL3774295	469
		Lysine-specific demethylase 5C	KDM5C	CHEMBL2163176	147
		Lysine-specific demethylase 6A	KDM6A	CHEMBL2069164	29
		Lysine-specific demethylase 6B	KDM6B	CHEMBL1938211	203
		Lysine-specific demethylase 7	KDM7A	CHEMBL2163177	35
	**LSD**	Lysine-specific histone demethylase 1	KDM1A	CHEMBL6136	1710
		Lysine-specific histone demethylase 1B	KDM1B	CHEMBL1938208	62
**BETi**	**Bromo and Extra terminal Domain**	Bromodomain-containing protein 1	BRD1	CHEMBL2176774	121
		Bromodomain-containing protein 2	BRD2	CHEMBL1293289	570
		Bromodomain-containing protein 3	BRD3	CHEMBL1795186	474
		Bromodomain-containing protein 4	BRD4	CHEMBL1163125	4864
		Bromodomain testis-specific protein	BRDT	CHEMBL1795185	119

The quest for finding epigenetic inhibitors led to the **first generation of epidrugs,** characterized by a meager degree of selectivity (57). Epidrugs of the first generation include DNMTi and HDACi, some of which have already been approved to treat hematological malignancies (58). DNMTi are pyrimidine analogs incorporated into DNA during replication and form covalent DNA adducts that cause DNA damage response activation and eventually lead to apoptosis. This was not without cytotoxic implications (3, 59). On the other hand, first generation HDACi are molecules that inhibit the Zn^2+^ dependent HDAC enzymes, except for sirtuin inhibitors, which inhibit a specific class of histone deacetylases that depend on NAD+ to perform their catalytic activity (59).

First-generation inhibitors represented many undesirable pharmacokinetic properties and poor target selectivity, resulting in the need for the creation of **second-generation** **epidrugs**, which included DNMTi (such as zebularine and guadecitabine), and HDACi (including hydroxamic acid, belinostat and panobinostat, tucidinostat and valproic acid) with improved physiological properties (59).

The second generation of epidrugs was characterized by strong academic research accompanied by industrial drug discovery to find molecules that resembled first generation epidrugs. The hypothesis was that molecules with more potent inhibitor action and fewer side-effects could be found. Another thing to consider was pharmacokinetics: first generation epidrugs had poor bioavailability, were more active within non pH physiological ranges, and were targets of cellular deaminases, which ultimately meant a short half-life for these compounds.

Ultimately, the **third generation of epidrugs** reflected that epigenetic factors could write, delete, or read epigenetic marks in the form of protein complexes. Therefore, a deeper understanding of epigenetic protein’s interactome is essential for the design of highly selective epidrugs (57). Epi-drugs of third generation includes, among others, histone methyltransferase inhibitors (HMTi), histone demethylase inhibitors (HDMi), and bromodomain and extra-terminal domain inhibitors (BETi) (59).

### DNMT Inhibitors

**Figure 3:**
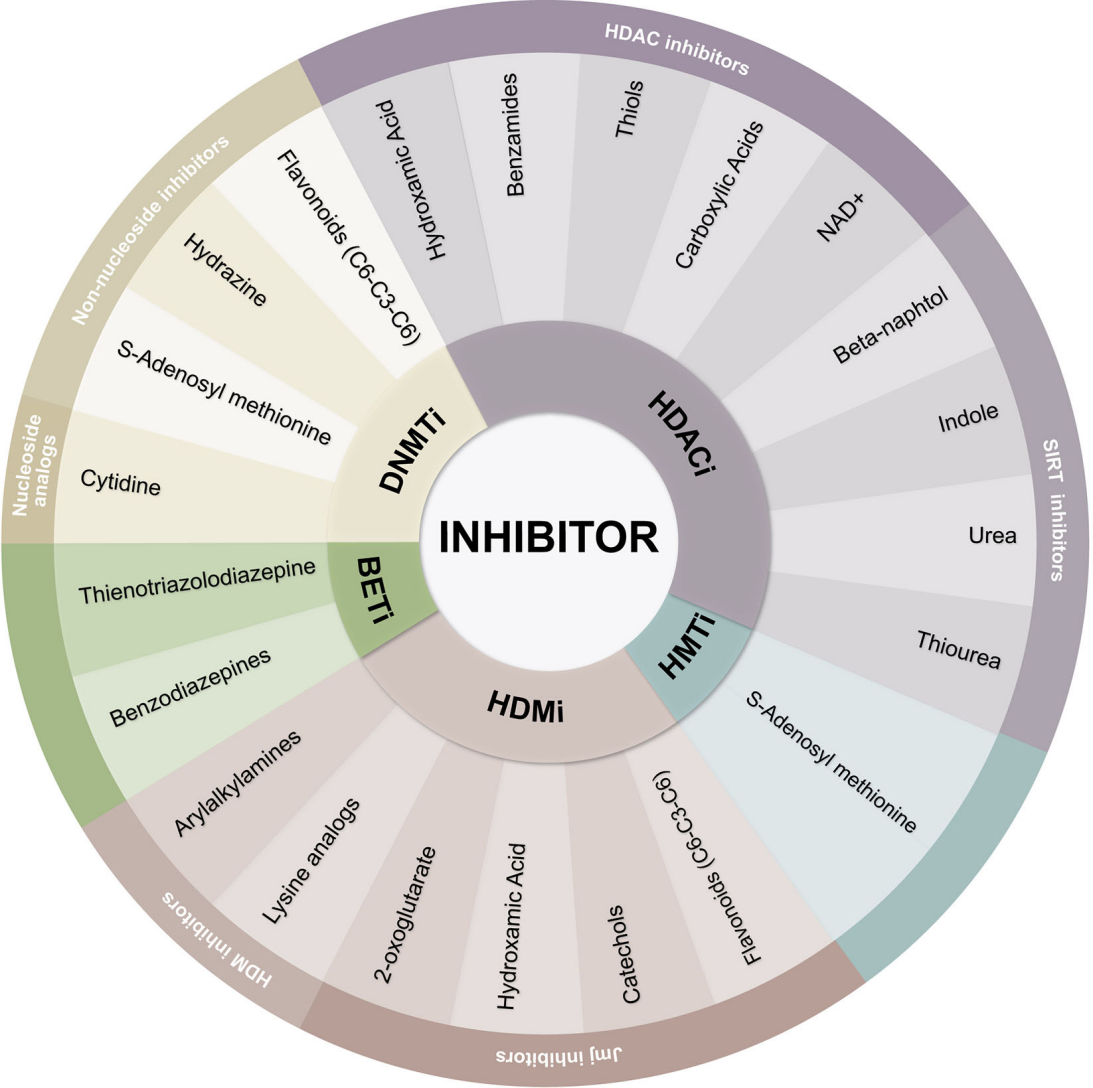
Classification of epigenetic inhibitors. Epigenetic inhibitors are classified as DNMTi, HDACi, HMTi, HDMI, and BETi. The chemical nature of each inhibitor defines the affinity of its targets.

DNA methylation inhibitors intercalate between DNA base pairs and suppress the CpG dinucleotide’s methylation, especially important at CpG islands. These inhibitors can be classified as DNMTi nucleoside analogs and non-nucleoside analogs (60) (**Figure 3**). DNMTi cytidine analogs are usually chemically unstable, and because of their similarity to cytidine, DNA and RNA polymerases identify both compounds and add them into growing nucleic acid chains, therefore hampering their selectivity (61).

Since the first DNMTi discovery (azacytidine), the number of inhibitors of DNMT has increased exponentially. The CHEMBL database reports 841 compounds tested for DNMT1 inhibition (CHEMBL1993), 258 compounds for DNMT3A (CHEMBL1992), and 80 compounds for DNMT3B (CHEMBL6095) (62) (**Table 1**, DNMTi section).

Among azacytidine derivatives, 5-aza-2’-deoxycytidine gained importance in the clinic, commonly known as “Decitabine”. Decitabine contains DNA sugar deoxyribose and is only integrated into DNA, while azacytidine allows for both RNA and DNA incorporation (14). Of note, Azacitidine and decitabine have both the same action mechanism. They both behave as a suicide substrate, trapping DNMTs after metabolic conversion and incorporation into DNA (3).

Guadecitabine is a hypomethylating agent of the second generation whose active metabolite is decitabine. Guadecitabine holds an amazing property: it is not a cytidine deaminase substrate, thus improving its selectivity. This drug has shown promise in treatments and recently tested in a Phase II clinical trial for treating non-intensive chemotherapy candidates with AML (63).

In 2004, azacytidine became the first medication approved by the FDA for all stages of myelodysplastic syndrome, a bone marrow disorder with a high risk of AML progression, characterized by irregular blood cell development, followed by decitabine in 2006 (64). These two drugs are currently used as first-line MDS therapy when other therapies are insufficient (14) (**Table 2**, DNMTi section).

**Table 2:** Overview of epigenetic inhibitors currently in clinical trials for cancer therapies.

Inhibitor	Mechanism of Action	Functional Molecule or Chemical Group	Examples	CAS	Clinical Trials				
					Phase Studies				Conditions
					I	II	III	IV	
**DNMTi**	Nucleoside analogs: Cytidine analogs incorporate into DNA instead of cytidine, covalently linking the enzyme and leading to DNMT degradation	Cytidine	Azacytidine	320-67-2	272	350	58	7	MDS, CML, AML, glioma, prostate cancer, pancreatic cancer, ovarian cancer, metastatic melanoma.
			Decitabine	2353-33-5	189	240	51	7	CML, AML, MDS, prostate cancer, thyroid cancer.
			Guadecitabine	929901-49-5	15	23	3	0	AML, MDS, HCC, CMML, ovarian cancer, urothelial carcinoma, colorectal cancer, peritoneal cancer
			5-fluoro-2’-deoxycytidine	10356-76-0	3	1	0	0	AML, MDS, Head and Neck Neoplasms, Lung Neoplasms, Urinary Bladder Neoplasms, Breast Neoplasms
			4’-thio-2’-deoxycytidine	134111-30-1	2	0	0	0	Currently establishing the safety, tolerability, and MTD in patients with refractory solid tumors.
	Non-nucleoside inhibitors either block the DNMTs enzyme catalytic site, interact with enzyme recognition of target sequences or are SAM cofactor competitors.	S-Adenosyl methionine	Sinefungin	58944-73-3	0	0	0	0	NA
		Hydrazine	Hydralazine	86-54-4	6	16	13	12	ovarian cancer, cervical cancer, refractory solid tumors, breast cancer.
		Flavonoids (C6-C3-C6)	Epigallocatechin-3-gallate	989-51-5	18	44	14	3	Adenocarcinoma of the prostate, head and neck cancer, colon cancer, pancreatic cancer, breast cancer, lung cancer, bladder cancer, colorectal cancer, prostate cancer.
**HDACi**	HDACi are molecules capable of Zinc trapping that bind to the zinc-containing catalytic domain of HDACs and supress their deacetylase enzymatic activity	Hydroxamic Acid	Vorinostat	149647-78-9	165	149	9	0	Rhabdomyosarcoma, Leiomyosarcoma, Lymphoma, melanoma, Lung carcinoma, lung cancer, head and neck cancer, leukemia, breast cancer, MDS, ovarian cancer, glioblastoma, pancreatic cancer, breast cancer.
			Trichostatin A	58880-19-6	1	0	0	0	Relapsed or Refractory Hematologic Malignancies
			Belinostat	866323-14-0	32	25	0	0	MDS, Non-Hodgkin lymphona, mantle cell lymphoma, diffuse large B-cell lymphoma, breast cancer, ovarian cancer, lung cancer, glioblastoma, AML, ATLL, bladder cancer, liver cancer,
			Panobinostat	404950-80-7	87	78	7	1	AML, MDS, lung cancer, gliosarcoma, prostate cancer, multiple myeloma, CMML, breast cancer, pancreatic cancer.
			dacinostat	404951-53-7	0	0	0	0	NA
			givinostat	497833-27-9	5	15	2	0	chronic lymphocytic leukemia, multiple myeloma, hodgkin’s lymphoma.
		Benzamides	Entinostat	209783-80-2	40	37	2	0	breast cancer, prostate adenocarcinoma, renal cell carcinoma, lymphoma, MDS, melanoma, lung cancer, AML, colorectal cancer, pancreatic cancer
			mocetinostat	726169-73-9	14	15	0	0	urothelial carcinoma, Hodgkin lymphoma, Head and Neck cancer, MDS, lung cancer, melanoma.
		Thiols	Romidepsin	128517-07-7	55	57	5	0	T cell lymphoma, glioma, multiple myeloma, CTCL, leukemia, astrocytoma, pancreatic cancer, lung cancer, thyroid cancer, prostate cancer, male breast cancer, renal cancer, bladder cancer.
		Carboxylic Acids	Valproic acid	1069-66-5	85	115	90	89	AML, MDS, Head and Neck cancer, SCC, glioma, bladder cancer, sarcoma, glioblastoma, leukemia, breast cancer, lung cancer.
			Butyric Acid	107-92-6	1	3	2	0	schyzofrenic disorders
			Phenylbutiric Acid	1821-12-1	20	30	3	2	colon cancer, leukemia, gastric cancer, MDS.
			Pivanex	122110-53-6	1	3	0	0	melanoma, lung cancer, leukemia.
	SIRTi are small molecules, many of them recently discovered by cell-based screening assays, with multiple inhibition mechanisms including reactivity with chemical intermediates, non-competitive inhibition with substrate and uncompetitive inhibition with NAD+.	NAD+	Nicotin	54-11-5	0	0	0	0	NA
		beta-naphtol	sirtinol	410536-97-9	0	0	0	0	NA
			splitomicin	1384339	0	0	0	0	NA
			salermide	1105698-15-4	0	0	0	0	NA
			cambinol	14513-15-6	0	0	0	0	NA
		indole	EX-527	49843-98-3	0	1	0	0	Endometriosis
			oxyndole	59-48-3	0	0	0	0	NA
		urea	suramin	129-46-4	8	12	3	0	lung cancer, breast cancer, adrenocortical carcinoma, renal cancer, prostate cancer, bladder cancer, multiple myeloma, head and neck cancer.
		thiourea	tenovin	380315-80-0	0	0	0	0	NA
**HMTi**	HKMTi are SAM like molecules and molecules that directly inhibits the enzyme S-adenosyl-L-homocysteine hydrolase or interact with the cofactor binding pocket of KMTs	S-Adenosyl methionine	Sinefungin	58944-73-3	0	0	0	0	NA
			EPZ004777	1338466-77-5	0	0	0	0	NA
			EPZ-5676	1380288-87-8	4	2	0	0	AML, MDS, leukemia
			EPZ004777	1338466-77-5	0	0	0	0	NA
			Valemetostat	1809336-39-7	1	1	0	0	leukemia, lymphoma, prostate cancer, renal cancer.
			tazemetostat	1403254-99-8	11	10	2	0	B cell lymphoma, prostate cancer, mesothelioma, Non Hodgkin lymphoma, tissue sarcoma, Bladder cancer, sinonasal carcinoma, follicular lymphoma.
	Most HRMT inhibitors are molecules which occupy and inhibit the SAM pocket, the substrate pocket, or both.	S-Adenosyl methionine	GSK3326595	1616392-22-3	2	0	0	0	neoplasms
**HDMi**	HDM inhbitors are molecules that inhibit monomine oxidases family of enzymes or that are substrate mimics (lysine analogs).	Arylalkylamines	Phenelzine	51-71-8	4	2	0	0	breast cancer, prostate cancer.
			Tranylcypromine	155-09-9	6	3	1	3	AML, MDS
			Pargyline	306-07-0	0	0	0	0	NA
		Lysine analogs	propylhydrazine	5039-61-2	0	0	0	0	NA
	JmjC inhibitors are derivates of 2OG, hydroxamic acids, catechols and flavonoids.	2-oxoglutarate	N-oxalylglicine	5262-39-5	0	0	0	0	NA
		Hydroxamic Acid	Methylstat	1310877-95-2	0	0	0	0	NA
		Catechols	Hematoxylin	517-28-2	0	0	0	0	NA
			Caffeic acid	331-39-5	3	1	3	1	esophagus cancer
		Flavonoids (C6-C3-C6)	Myricetin	529-44-2	0	0	0	0	NA
			Baicalein	491-67-8	0	2	0	0	Influenza
			Epigallocatechin-3-gallate	989-51-5	18	44	14	3	Adenocarcinoma of the prostate, head and neck cancer, colon cancer, pancreatic cancer, breast cancer, lung cancer, bladder cancer, colorectal cancer, prostate cancer.
**BETi**	BET inhibitors are derivates of benzodiazepines that take up the hydrophobic región of BET enzymes which binds acetylated lysines.	Thienotriazolodiazepines	JQ1	1268524-70-4	0	0	0	0	NA
			CPI-203	1446144-04-2	0	0	0	0	NA
			OTX015	202590-98-5	5	2	0	0	AML, glioblastoma, breast cancer, lung cancer, prostate cancer.
		Benzodiazepines	CPI-0610	1380087-89-7	3	2	0	0	Myeloma, lymphoma, leukemia, MDS.
			Molibresib	1260907-17-2	2	1	0	0	lymphoma, NUT carcinoma,

As mentioned before, DNMTs have two substrates, the methyl group donor cofactor SAM and the methylated cytosine. Non-nucleoside DNMTi includes analogs of the methyl donor S-adenosyl-L-methionine (SAM) and small molecules that interact with the active site of the enzyme DNMT (**Figure 3**). Indeed, it is possible to obtain potent DNMT inhibitors by designing substrate analogs and connecting them (65). This strategy has resulted in the most effective way to inhibit DNMTs and reactivate genes in cancer cells by promoting demethylation (60). Many forms of these derivatives have shown remarkable results in many models of cancer and other human diseases. These include hydralazine, EGCG, RG108, MG98, and disulfiram (66–71) (**Table 2**, DNMTi section). MG98 is a second-generation phosphorothioate antisense oligodeoxynucleotide that inhibits translation effects of DNMT1 mRNA but has no apparent impact on tumors (72).

Despite preclinical evidence indicating a potentiating chemotherapy cytotoxic activity of HDAC inhibitors and DNMT inhibitors, clinical outcomes have been discouraging: three of the five main combination randomized trials were stopped because of ineffectiveness or disadvantaged toxicity profiles compared to chemotherapy alone (59). The possible role of DNMT inhibitors remains unclear, but in conjunction with other therapies, these agents may theoretically still be of use.

There is a good scientific justification for combining DNMT inhibitors with HDAC inhibitors since both hypermethylated DNA and hypoacetylated histones are associated with closed chromatin states that repress gene expression by independent mechanisms. Further studies should be carried out into the efficacy of this combination at different dosages and durations of treatment. To date, hundreds of clinical trials have studied the effects of anti-DNA methylation therapy on different cancers.

### HDAC Inhibitors

The development of the first HDACi commenced with the finding that erythroleukemia murine cells differentiated in the presence of dimethyl sulfoxide (DMSO). Later, chemical analogs that could make similar interactions as DMSO were studied (56). This was the case of vorinostat (SAHA), a molecule capable of metal coordination and hydrogen bonding. Interestingly, natural compounds inhibitors of HDACs (trichostatin A and trapoxin A) were found to chemically resemble vorinostat at the hydroxamic acid moiety. The mechanism of action of these compounds inhibits HDACs by reversibly binding to Zn^2+^ in the enzyme’s active site. Since the discovery of vorinostat, a lot of new activity assays are performed every day with inhibitor compounds (62) (**Table 1**, HDACi section).

Zinc binding is essential for the inactivation of most HDACs (56). As mentioned before, the Zn-binding hydroxamic moiety has proven to be one of the most successful inhibitors, and thousands of synthetic HDAC inhibitors with this moiety have been reported. Many of these inhibitors have focused primarily on optimizing the pharmacokinetics of vorinostat and trichostatin A (**Figure 3**; **Table 2**, HDACi section).

Currently, vorinostat therapy clinical applications have been applied to neurological conditions and, surprisingly, to reactivating chronic viral infection (73). Therapies for HIV-1 patients do not kill the virus entirely because it may be latent in reservoirs of CD4 + cells (74). Epigenetic mechanisms regulate viral latency, and so, clinical trials to test the effect of vorinostat therapy in reactivation of HIV-1 viral latency are currently being performed.

This optimizing focus led to the design of the hydroxamic acid containing HDACi, such as belinostat, dacinostat, givinostat, and panobinostat. The latter being the only HDACi with approval within the EU. As single agents, these molecules have shown limited efficacy, but when in combination with DNMTi, they have shown to be more effective, especially in patients with solid tumors (75, 76). Other metal-binding functional groups have been of great interest to this group. This is the case of thiols, benzamides, and carboxylic acids (56). Examples of these functional groups can be found in the drugs: romidepsin, entinostat, mocetinostat, and short-chain fatty acids, such as sodium butyrate, Pivanex, phenylbutyric acid, and valproic acid (**Figure 3**; **Table 2**, HDACi section).

Unlike hydroxamic acid analogs, short-chain fatty acids occupy an acetate escape tunnel, which may have a zinc-binding function or compete with an acetate group released in the deacetylation reaction. These are the least potent type of HDACi (77). The benzamide inhibitor class consists of a chemical moiety capable of contacting specific amino acids in the HDAC core tube active site, with or without zinc ion binding (78). These inhibitors are active at micromolar levels. The antiproliferative and cytotoxic activity has been shown by entinostat against several tumor cell lines *in vitro*. Entinostat is a clinical trial available orally active inhibitor (79) (**Figure 3**; **Table 2**, HDACi section).

Currently, the discovery of sirtuin inhibitors (SIRTi) is an ongoing quest in which most compounds are still under preclinical investigation (80). Most efforts have been driven toward the discovery of SIRT1 and SIRT2 inhibitors. SIRT1 inhibitors have been proposed for treating cancer, for they have shown to inhibit TNBC cell growth, survival, and tumorigenesis (56, 81). Nicotinamide is the only inhibitor of sirtuin currently used in solid tumor clinics (82). SIRTi can be categorized as β-naphthols (sirtinol, splitomicin, salermide, and cambinol), indoles (EX-527 and oxindole), and urea (suramin and tenovin) (83) (**Figure 3**; **Table 2**, SIRTi section).

HDACi have many biological effects due to changes in patterns of histone acetylation and many non-histone proteins, including proteins involved in gene expression control, extrinsic and intrinsic apoptosis pathways, the progression of the cell cycle, redox pathways, mitotic division, DNA repair, cell migration and angiogenesis (56). Whether selective inhibition of HDACs will be beneficial as anti-cancer agents over broader-acting HDACi is a question that remains unanswered (56).

### Histone Methyltransferase Inhibitors

HMTs are enzymes that add up to three methyl groups to lysine (KMTs) or arginine (RMTs) residues in histone proteins (84). Lysine methylation may either activate or silence gene transcription depending on the lysine residue involved (85). Nearly 100 KMTs have been described which use the SAM molecule as the methyl donor (14). SAM-like molecules, such as sinefungin, compete with SAM for its binding site (**Figure 3**). These molecules are inhibitors of all SAM using enzymes, like HMTs (14). KMT drug discovery heavily relies on their cofactor binding pocket, which has structural characteristics convenient for inhibitor interaction and makes these enzymes appealing for the design of small molecular inhibitors for interference (80). Examples of HMTi can be found in drugs such as EPZ004777, EPZ-5676, DZNep, pinometostat, and tazemetostat. Pinometostat and tazemetostat are selective DOT1L and EZH2 inhibitors, respectively (**Table 2**, HMTi section).

Both inhibitors are of interest in some types of cancer because DOT1L is a KMT involved in abnormal methylation of H3K79 and expression of HOX genes that cause leukemia (Copeland et al., 2013), while elevated expression of the KMT, EZH2, is associated with many forms of cancer due to hypermethylation of H3K27 which facilitates transcriptional silencing (80). Also, in B-cell-lymphoma patients, EZH2 mutations occur with a frequency of approximately 15-20 percent in either tumor type, particularly in diffuse large-B cell-lymphomas and follicular lymphomas (86, 87). These modifications contribute to the more effective trimethylation of H3K27 by the mutant form of this protein (88). Preclinical studies showed that EZH2 inhibitors induced the arrest of proliferation, differentiation, and eventual apoptosis of DLBCL cells. These results were stronger in DLBCL cells that bear EZH2 mutations, but they also occurred in EZH2-wild-type DLBCL cells (89).

While several small molecule inhibitors have been developed for PRMTs with adequate potency, most PRMT inhibitors’ selectivity remains to be improved. Therefore, the detection of PRMT inhibitors involves further analysis of novel approaches (i.e., allosteric control) (90). Three PRMT inhibitors, including PRMT5 inhibitor GSK3326595 (**Table 2**, HRMTi section), and JNJ-64619178 as well as PRMT1 inhibitor GSK3368715 have entered clinical trials so far. PRMT inhibitors with novel action mechanisms and strong drug-like properties will shed new light on developments in drug discovery and development of PRMTi (87, 90). The number of inhibitor assays reported on CHEMBL database against the enzymatic activity of the HMTs increases everyday (62) (**Table 1**, HMTi section).

### Histone Demethylase Inhibitors

Significant progress has been made in the development of JmjC-KDM inhibitors since the first inhibitors were identified in 2008 (91). The vast majority enter the catalytic domain and inhibit the enzyme’s activity by chelating the active site Fe (II), interfering with the 2OG binding. Because of the similarity between JmjC-KDMs’ active site pockets, it has proved difficult to achieve selectiveness in the broad superfamily of 2OG dioxygenases (92). The recent availability of JmjC-KDM crystal structures has encouraged medicinal chemistry efforts and has made it possible for the JmjC-KDMs to produce many chemical candidates. Examples of these inhibitors include hydroxamate derivatives, pyridinedicarboxylate derivatives, N-oxalyl amino acid derivatives, and agents which interfere with metal binding (71) (**Figure 3**; **Table 2**, HDMi section).

In 2004, Professor Yang Shi first described LSD1 and discovered that it had significant biological functions in a wide variety of biological processes, including cancer (93). During carcinogenesis, in AML and SCLC, elevated levels of LSD1 were observed (94). Pharmacological LSD1 inhibition with small molecules has shown that it suppresses the division, proliferation, invasion, and migration of cancer cells (95). LSD1 thus becomes an evolving clinical target for anticancer therapy. Many LSD1 inhibitors, including natural products, peptides, and synthetic compounds, have been identified.

The similarity of LSD demethylases with monoamine oxidases (MAOs) has started the quest for repurposing MAO inhibitors to find inhibitors for these types of enzymes. Initially approved by the FDA for the treatment of mood and anxiety disorders (96), the MAO inhibitor tranylcypromine (TCP) was found to be able to inhibit its homolog LSD1 moderately by forming covalent adducts (97). As a result, many MAO inhibitors (MAOi) such as pargyline, phenelzine, and tranylcypromine have been shown to inhibit HDM KDM1A (80) (**Figure 3**; **Table 2**, HDMi section). New studies are now ongoing in clinical trials with some TCP-based LSD1 inhibitors alone or combined therapy with other therapeutic agents for treating cancer (98).

### Bromo and Extra Terminal Domain Inhibitors

Bromodomains are protein motifs present in several epigenetic readers including BET family, that recognize and bind to acetylated lysine residues located on histone tails. BETs consist of two bromodomains and an extra-terminal region. The BET family includes the Bromodomain testis-specific protein (BRDT), BRD2, BRD3, and BRD4 (99). BETs lead to malignancies production and progression by stimulating and enhancing the expression of main oncogenes such as *MYC* (100). Indeed, when treated with the inhibitor JQ1, BET inhibition resulted in *MYC* downregulation, which resulted in decreased levels of mRNA and protein in mouse MLL-fusion leukemia cells (101).

In various forms of cancers, including breast, neuroendocrine, ovarian, rhabdomyosarcoma, and glioma, preclinical studies of BET inhibitors have shown their efficacy (87). They disrupt the recognition by BET-containing reader proteins of acetylated lysine residues in histones, a mark associated with active transcription (102). The mechanism of BETi relies on the fact that the region that binds acetyl-lysine is hydrophobic and can be taken up by small hydrophobic molecules that specifically target this catalytic site. Examples of these inhibitors can be found in Thienotriazolodiazepines (JQ1, CPI-203, OTX015) and Benzodiazepines (CPI-0610 and molibresib) (**Figure 3**; **Table 2**, BETi section).

Preliminary clinical trials have demonstrated that BET inhibitors cannot induce long-lasting cytotoxic effects in human cancers when administered as single agents (103). Nevertheless, the potential of combinations with other epigenetic therapies is important (104). Although BET inhibitors’ toxicity may reduce such combinations, HDACi studies indicate that combinations with reduced doses may be effective, possibly reducing toxicity. This also reflects on the number of inhibitor assays for BRDs (62) (**Table 1**, BETi section).

### The Basis for Drug Repurposing

**Figure 4:**
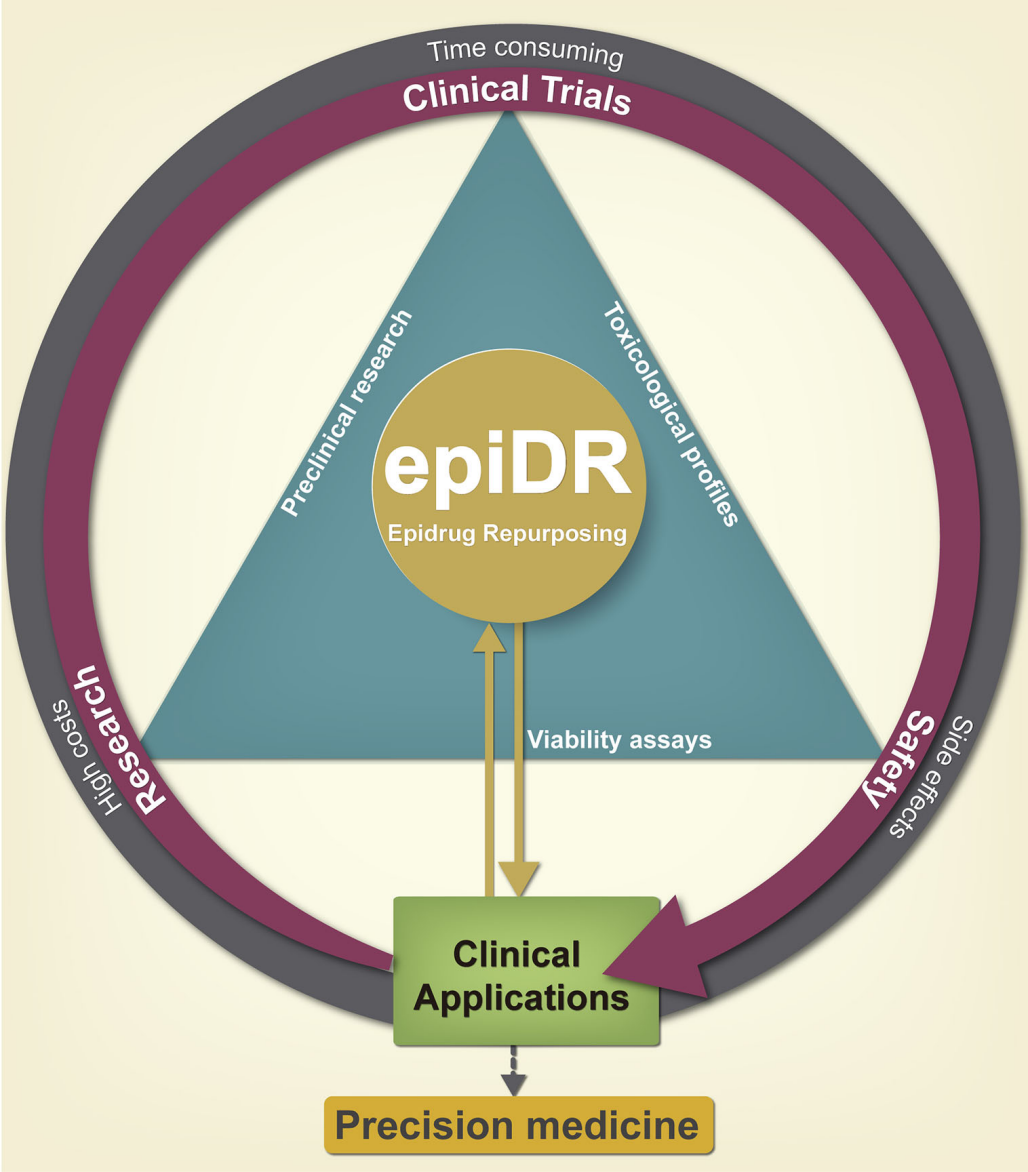
Advantages of pharmacological epi-drug repurposing in clinical applications. Drug repurposing serves as a shortcut reducing the time of incorporating a drug into the clinic; since the preclinical phase has already been carried out previously, giving a second chance to old drugs. Initially, it reduces the cost of development and toxicity research, which leads to greater cost-benefit efficiency for the pharmaceutical industry by generating a new cancer therapy. The repositioning of epi-drugs is a promise for the generation of new drugs of precision medicine.

Although epigenetic therapy has proven to be remarkably effective, epidrug discovery remains as a traditional “*de novo*” drug discovery pathway, which has significant disadvantages such as high costs, time consuming, and low success rate (105, 106) (**Figure 4**). An answer that addresses these problems and could speed up epidrugs in the clinic has arisen from the relatively recent idea of using known drugs for new targets, commonly known as drug repurposing (DR). This approach has gained considerable popularity, emerging as an interesting approach in cancer therapy research and many fields within medicine (107).

DR is the discovery process of finding new medical uses of a preexisting drug which was previously approved for another indication, withdrawn from the market due to adverse effects or disapproved for failing to prove its efficacy and safety (11, 107) (**Figure 4**).

This approach includes the selection of drugs with promising repurposing potential and it also has important advantages over the “*de novo*” drug discovery processes. Previously assessed drug safety significantly reduces both costs and time for making these drugs readily available for use in the clinic (108, 109).

Historically speaking, repurposing of medications was mainly fortuitous; if an off-target effect or newly discovered target was detected, it was sure for it to be targeted for commercial usage. Examples of this are shown in drugs like sildenafil citrate, whose repurposing for erectile dysfunction was not based on a systemic approach, nor was thalidomide repurposing for erythema nodosum leprosum (ENL) and multiple myeloma, which are still the most promising examples of DR (107). Sildenafil was first formulated as an antihypertensive medication. However, after Pfizer reprofiled it for erectile dysfunction therapy and sold it as Viagra, it held the lead market share in erectile dysfunction medications in 2012, with global sales totaling more than 2 billion (110). Thalidomide, an antiemetic first sold in 1957, was discontinued within four years due to its notorious association with teratogenic defects in infants born to mothers who took the drug during their first trimester of pregnancy (107). However, the efficacy of thalidomide, first in ENL and decades later in multiple myeloma has been successfully demonstrated. Ever since, thalidomide has achieved considerable market success for treating multiple myeloma and has also contributed to the production and authorization of many more effective formulations, such as lenalidomide, which had $8.2 billion in worldwide revenues in 2017 (111).

These achievements have led to the implementation of systematic approaches to detect repurposable substances (109). The field of DR is fascinating, and its importance reflects in the vast number of drug projects of pharmaceutical companies that already have several candidate molecules that, although successful in phase I, they did not prosper in Phase II or III clinical trials. This gives rise to the existence of several known molecules, which are relatively safe to use in the clinic. Hence, this large reservoir of molecules provides a vast niche for the search for repositionable drugs, which is much larger than the set of approved drugs (112).

A DR approach usually consists of three phases before the target drug is taken into further development: The selection of a target molecule for a specific indication, analysis of the drug impact in preclinical models, and the evaluation of the effectiveness in clinical trials in phase II, when enough adequate safety results are available from phase I tests. These methods can be classified into computational approaches and experimental approaches, which are now both being widely used synergistically. DR is encompassed within these two large fields, focused on clinical evidence (109).

Experimental approaches include **binding assays** for the identification of novel target interactions. These types of assays come from proteomic methods, like affinity chromatography and mass spectrometry are used to detect novel targets of existing drugs (113); and **phenotypic screening**, which are approaches based on *in vitro* or *in vivo* models of disease screening of compounds can indicate clinical potential (114). These approaches offer testing in a relevant biochemical context by performing *in vitro* assays with live cells (115, 116). The evolution of *in vitro* screening has led to systematize drug discovery, allowing ultra-high-throughput screening, analyzing up to 10,000 compounds per day (116, 117); however, the main limitation of these methodologies are the high costs of the required infrastructure, as well as nonspecific results (8).

Computational methods include the study of large sets of data (e.g., gene expression, chemical composition, genotype or proteomic data or electronic health records) that lead to the development of reprofiling hypotheses (118). Computational approaches include: **signature matching**, which results for comparing a drug signature such as its transcriptomic, structural or adverse effect profile to that of another pharmaceutical product or disease phenotype (119); **molecular docking**, a structural computational strategy focused to predict complementarity of the binding site between a drug and a receptor (120); **genetic association**, a high throughput analysis of genes associated with a disease which can turn out to be potential targets for drugs (121); **pathway mapping**, another approach that analyses biological pathways in order to develop networks of drugs or disorders based on patterns in gene expression, disease biology, protein interactions or GWAS data to better classify repurposable candidates (122); **retrospective clinical analysis,** a systematic review of electronic health records, data from clinical trials and surveillances post-marketing could be useful identifying repurposable drugs; and **novel sources**, which is the combination of large-scale in-vitro drug screens with genomic data, electronic health records and self-reported patient data represents new ways to repurpose drugs (123, 124).

In sum, these approaches allow multiple manners for conducting DR. However, these methodologies applications need to be taken with caution, as many of them seem to be reductionist (117, 125). Numerous strategies are now coupling drug networks with computational analysis to characterize different diseases’ metabolic pathways. These efforts aim to identify drugs acting not only on a single target but also on a whole network of proteins (126, 127). In every computational approach, experimental validation is compulsory since the actual methods are not 100% accurate.

HTS (High-Throughput Screening) is the most common approach in DR of epidrugs, and most of them are designed to inhibit catalytic sites of epigenetic writer enzymes (128). Computational methods, such as virtual screening, aim to efficiently discover novel active compounds against epigenetic factors (8). The increasing attention on epigenetic targets as an opportunity for DR provides high expectations. Next, we will summarize the current efforts in epidrug repurposing for cancer therapy.

### Available Databases Focused on Exploration and Recompilation of DR Research

Nowadays, there is a large amount of information available focused on the search and annotation of drugs to be repurposed and the drugs that currently have research that supports their proposed new uses. Some public databases such as ChemBL, DrugBank, and DrugCentral are repositories of bioactivity data and drug chemical structures. These databases summarize multiple indications and chemical drug-target interactions. More specifically, the FDA-approved epidrugs are gathered in several databases focused on tested epidrugs and provides information about annotation tools (**Table 3**, Section Epidrugs). These databases are useful because they facilitate the integration of epidrug datasets obtained from experimental and computational approaches, reducing the manual search of information, and helping to increase collaboration on the field.

**Table 3:** Some databases and tools that summarize the current knowledge on DR.

Category	Database name	Link	Key features	Reference
**Drug-target interactions and bioactivity databases**	ChEMBL	ebi.ac.uk/chembl/	Provides bioactivity data, structures and properties, clinical trials and drug annotations references for diseases	(62)
	PubChem	pubchem.ncbi.nlm.nih.gov/	Provides chemical structures and physical properties, bioactivity information, current patents, toxicity and safety; among others	(129)
	DrugCentral	drugcentral.org/	Provides chemical structures, chemical entities action, drug mode of action, dosage and pharmacological indications	(130)
	DrugTargetCommons (DTC)	drugtargetcommons.fimm.fi/	Bioactivity data, protein classification, assays and clinical trials data and disease gene associations for many proteins	(131)
	DrugBank	drugbank.ca/	Matches drug bioactivity information with drug-target physiological information	(132)
**Epigenetic drugs databases**	HEDD	hedds.org/index.jsp	Integration of experimental epigenetic drug datasets, provides information from target-disease, and tools from high-throughput screening	(133)
	HISTome2	actrec.gov.in/histome2	Provides histone proteins data and 127 epidrugs that have been categorized by modifier type; and advanced tools for histone modifier-drug prediction	(134)
	dbEM	crdd.osdd.net/raghava/dbem	Provides epigenetic modifiers data in normal and cancer genomes; and information for 54 drug molecules against different epigenetic proteins	(135)
**Drug Repurposing databases**	PROMISCUOUS	bioinformatics.charite.de/promiscuous	Provides an exhaustive set of drugs (25,000), experimental assays and annotations from protein relationships	(136)
	REPO Hub	clue.io/repurposing	Repurposing library that assemble a collection of 4,707 compounds, experimentally confirmed, clinical trials and annotations based on literature-reported targets	(137)
	RepurposeDB	repurposedb.dudleylab.org	Provides a summarize on drug repositioning studies reported on public databases. Assemble a repertoire of drugs, drug targets and associated disease indications	(138)
	repoDB	apps.chiragjpgroup.org/repoDB	Provides information from 1,571 compounds, both approved and failed drugs; as well as computational repositioning tools	(139)
	Project Repethio	het.io/repurpose	Provides a compilation of 3394 repurposing candidates based on computational predictions	(140)
**Drug Repurposing in cancer databases**	ReDO project	redo-project.org/	Provides a curated list of 270 drugs with pre-clinical and clinical evidence of anti-cancer action	(141)

Other databases that aim to summarize the current efforts and latest frontiers in DR research are the REPOHub, repoDB, and the Project Repethio; these include clinical trials, pre-clinical tools for annotations, and information resources. Unlike the previous ones, these databases focus on gathering and matching the results from both predictive tools and experimental or clinical trials, resulting in faster results on drugs that could be repurposed (**Table 3**, Section Drug Repurposing). Tanoli et al., 2020 summarize the types of data available through multi-database exploration focused on DR (142). Currently, the ReDO project (Repurposing Drugs in Oncology) is probably the only database focused on assembling DR for cancer targets. And it has played a crucial role in the development of research for new drugs to cancer therapy with the DR approach.

### Epidrug Repurposing in Cancer (Epi-DR)

The interest in oncological DR has emerged as a response to the declining productivity of oncological drug development (143) and as a source of low-cost treatments to meet the increased demands for novel treatments, in efforts to overcome chemoresistance and reduce the development time of *de novo* drugs (144).

Some widely used and well-known drugs for cancer therapy are examples of epi-DR, with an effect on epigenetic targets, and are either currently FDA-approved or under clinical development (145). The first repurposed drugs as an anticancer epidrug in the field were the 5-azacytidine and 5‐aza‐2′‐deoxycytidine (decitabine) (146). At first, these drugs were both approved by the FDA to treat myelodysplastic syndromes due to their antimetabolic effects on *in vitro* assays in cancer cells (146). However, the toxicity shown by 5-azacytidine led to other chemotherapeutic regimens being preferred (146); later, it was found that azacytidine and decitabine could both inhibit DNA methylation and were incorporated by tumor cells and also in myelodysplastic syndromes (146–148).

### DNMT Inhibitors

The natural compound **Harmine** downregulates the expression of DNMT1, which results in reactivation of the p15 tumor suppressor gene in AML. Future studies are expected to assess if Harmine can be considered a potential therapy for AML and if it can be used as a single agent or adjuvant (149). **Chlorogenic acid** is a polyphenol coffee that has been found to suppress DNMT1. Its inhibitory activity derives from a chemical change resulting in increased S-adenosyl-L-homocysteine (SAH) production. Chlorogenic acid has been shown to inhibit DNMT1, using breast cancer cell lines, which lowers DNA methylation (150).

**Laccaic acid A** is a direct, competing DNMT1 natural compound inhibitor that reactivates genes silenced by promoter DNA methylation synergistically with 5-azadC in breast cancer cells (151). **Procaine** is a promising treatment with growth-inhibiting and DNA-hypomethylation effects in cancer cells. Especially in gastric cancer where its antiproliferative and apoptotic effects have been proven (152). Its well-defined, safe use as a local anesthetic, with well-known pharmacology, should promote procaine to pre-clinical trials (153). **Procainamide**, a derivative of procaine, hinders the enzymatic activity of DNMT1 by directly reducing the enzyme affinity for both DNA and S-adenosyl-L-methionine. It would be important to analyze whether procainamide, a fairly stable non-nucleoside inhibitor of DNMT1, will prevent cancer from arising (154).

A computer-based search for similarities between a database of approved drugs and 5-aza-2’-deoxycytidine has recently been detected as an ideal candidate for DR. **Mahanine**, a plant derived alkaloid, was shown to induce DNMT1 and DNMT3B proteasomal degradation by inactivating Akt, which in turn restored RASSF1A expression in prostate cancer cells. Mahanine then represents a possible therapeutic agent for advanced prostate cancer when RASSF1A expression is inhibited (155).

**Hydralazine**, approved as an antihypertensive, is a non-nucleoside DNMTi that interacts with the binding domain of DNMTs, and can decrease DNMT1 and DNMT3A mRNA expression and protein levels in T cell leukemia cell lines (156). In advanced cervical cancer, bladder, and cervical cancer cell lines, respectively (157, 158), hydralazine induces DNA demethylation and decreases DNMT activity. Also, hydralazine, combined with magnesium valproate, is an opportunity to reverse imatinib resistance in patients with several malignancies, including lung (NCT00996060), cervical (NCT00404326), and locally advanced breast (NCT00395655) cancers, as well as different solid tumors which are refractory to current therapies (159–161) (NCT00404508). **Olsalazine,** an FDA approved anti-inflammatory agent, has proven its hypomethylating and very low cytotoxicity effects in cell-based screen tests (162).

**Mithramycin A**, an antibiotic with potent antitumor activity, binds to sequences of GC-rich or CG-rich DNA and upregulates tumor suppressor genes’ expression by reducing the methylation of their promoters through binding and depleting the DNMT1 protein in lung cancer cells (163). **Nanaomycin A**, an anthracycline antibiotic, has demonstrated selectivity to DNMT3B in biochemical assays. Dock modeling strategies suggest that nanaomycin A is capable of binding DNMT3B’s catalytic site. Treatment of the human tumor lines of the colon, lung, bone marrow with nanaomycin A demonstrated substantial genomic demethylation. While it is unclear if anthracyclines will be a successful choice for clinical DR due to certain long-term cardiotoxicity concerns, Nanaomycin A is the first non-SAH DNMT3B-selective compound that offers valuable biochemical properties for additional studies (164).

**Disulfiram** is an alcohol aversive drug that has been approved by the FDA for more than 60 years for treating alcohol abuse. It allows acetaldehyde to accumulate in the blood by inhibiting ALDH (165). Disulfiram’s anticancer activity is mediated by its ability to suppress DNMT1 and through the reactivation of epigenetically silenced genes such as *APC* and *RARB* in prostate cancer cell lines (70) (**Table 4**, Section 1).

**Table 4:** Current DNMTi and DNMT-HDAC dual inhibitors repurposed drugs with applications in cancer therapy [*modified from Moreira-Silva et al. (9)].

Class	Compound	First indication	Epigenetic target	Drug-target interaction	Cancer model/New indication	Key features in mechanism	References
**Section 1. DNMT inhibitors**							
**Non-nucleoside analogs**	**Hydralazine**	Anti-hypertensor	DNMT1	Four high-affinity interaction points with DNMT1 through the residues Lys 162 and Arg 240 within the enzyme active site.	T‐Cell Leukemia cells	Increases LFA-1 expression inhibits T Cells ERK pathway phosphorylation, decreases DNMT enzyme activity, and decreases DNMT1 and DNMT3A protein levels. Reduces *de novo* methylation due to greater affinity to hemi methylated substrates (target of DNMT1),.	(156, 166)
					Breast Cancer cells	*In vivo* induces DNA demethylation and increases expression of ER as well as RARb, p12 and p16 *in vitro*.	(167)
					Bladder Cervical Cancer cells		(157)
					Prostate Cancer cells	Increases apoptosis, inhibits RGFR pathway, thus induces cell cycle arrest. Decreases DNMT1, DNMT3a and b protein levels. Upregulates p21 which decreases promoters DNA methylation and induces histone acetylation.	(156)
					Cervical Cancer cells	Induces APC expression, inhibits cell growth, induces cell cycle arrest and apoptosis. Promotes DNA demethylation.	(158)
	**Disulfiram (DSF)**	Alcohol aversive	DNMT1	DSF could interfere with the catalytic activity of DNMT1 by reacting with a citosine ring via thiol group of catalytic site of DNMT1.	Prostate Cancer cells	Reduces global 5mC content, through inhibition of DNMT1 activity on hemimethylated substrates. Decreases methylation in APC and RARB gene promoters, thus increasing re-expression. Inhibits growth and clonogenic survival of prostate cancer cell lines.	(70)
	**Procainamide**	Cardiac arrythmias	DNMT1	Partially competitive inhibitor of DNMT1 that interacts with the binding pocket of the enzyme	Prostate Cancer cells	Promotes GSTP1 CpG island hypomethylation, thus induces GSTP1 re-expression in LNCaP cells *in vitro* and in *in vivo* assays	(168)
					Breast Cancer cells	Induces DNA demethylation increases expression of ER RARb; also induces re-expression of p12 and p16 (*in vitro*).	(169)
					Colon Cancer cells	Greatly reduces affinity for hemi-methylated DNA and SAM in catalysis, reduces global 5mC content, thus reduces gene-specific hypermethylation at promoter CpG islands.	(154)
					Non-small Cell Lung Cancer cells	Inhibits DNMT activity and decreases promoter demethylation of WIF-1, restoring WIF-1 expression, thus downregulating the Wnt pathway	(169)
	**Procaine (PCA)**	Anesthesic for spinal block	DNMT1, DNMT3A	Interacts with the binding pocket of the enzyme inhibiting catalytic activity (non-nucleoside),	Breast Cancer cells	Demethylates densely hypermethylated CpG islands, reduces 5mC DNA content by 40%, restoring gene expression of RARβ2, and has growth-inhibitory effects, causing mitotic arrest	(153)
					Gastric Cancer cells	Inhibits DNMT1 and 3A activity through molecular docking in the catalytic binding site, disrupting the binding of DNMT to DNA. Reduces proliferation, induces apoptosis, and restores expression of CDKN2A and RARb	(152)
					Hepatocellular Carcinoma cells	DNA demethylation and silenced gene reactivation of p16, HAI-2/PB, and NQO1. Promotes cell cycle arrest and reduces viability. Also shown significant reduction in tumor volume *in vivo*.	(170)
					Non-small Cell Lung Cancer cells	Inhibits DNMT activity, causing promoter demethylation of WIF-1, thus restores WIF-1 expression and downregulation of Wnt pathway	(169)
**Antibiotic**	**Nanaomycin A**	Anthracycline antibiotic	DNMT3B	Interaction with active site of DNMT3B in specific a.a. (Glu697 Arg731 Arg733) of enzyme binding pocket, thus promoting a molecular docking in DNMT3B that inhibits enzymatic activity	Colon Cancer cells	Decreases DNMT1, 3A, 3B expression. Inhibits DNMT3B activity promoting reactivation of RASSF1A. Reduces cell proliferation and viability	(164)
					Lung Cancer cells		
					Bone marrow cells		
	**Mithramycin A (MMA)**	Hypercalcemia drug, and antineoplastic agent	DNMT1	Possibly interferes with DNMT1 binding at the CpG region in TSG promoters through binding DNMT1 protein or might be a form a complex between MMA, DNMT1 and double-stranded DNA	Lung Cancer cells	Inhibits DNMT1 activity and decreases protein level. Decreases CpG methylation on SLIT2 and TIMP-3 promoters, inducing re-expression. Inhibits invasor pehotype thus prevents metastasis	(163)
**Polyphenol**	**Chlorogenic acid**	Natural Compound (not approved)	DNMT1	Increases SAH formation inhibiting DNA methylation through COMT mechanism (non-competitive),	Breast Cancer cells	Inhibits DNMT1 activity, reduces methylation of the promoter region of the RARb gene	(150)
	**Harmine**	Natural Compound (not approved)	DNMT1	Not described	Myeloid Leukemia cells	Decreases DNMT1 gene expression, induces p15 promoter demethylation. Also decreases proliferation and promotes cell cycle arrest in G0/G1 phase	(149)
	**Laccaic acid A (LCA)**	Natural Compound (not approved)	DNMT1	DNA-competitive DNMT inhibitor through competition for the oligonucleotide substrate	Breast Cancer cells	Inhibits directly DNMT1, also have effects on DNMT3A, 3B inhibition, and reactivates genes silenced by promoter methylation (CEACAM5, DHRS3, RGS16)	(151)
	**Mahanine**	Natural Compound (not approved)	DNMT1, DNMT3B	Induces proteasomal degradation of DNMT1 and DNMT3B	Prostate Cancer cells	Inhibits DNMT activity, increases expression of RASSF1A and inhibits cyclin D1. Induces proteosomal degradation on DNMT1 and DNMT3B through Akt inactivation, thus facilitates demethylation of RASSF1A promoter and increases its expression	(155, 171)
	**Genistein**	Natural compound; isoflavone	DNMT	Inhibits DNA methyltransferase activity in a substrate- and methyl donor–dependent manner	Esophageal Squamous Cell Carcinoma cells	Promotes reversed DNA hypermethylation and reactivation of RARbeta, p16INK4a and MGMT trhough demethylation of promoter genes. Also inhibites cell growth	(172)
					Prostate Cancer cells	Induces DNA hypomethylation and reactivation of RARbeta	
					Breast Cancer cells		
					Renal Cancer cells	Inhibits DNMT1 activity, thus induces demethylation of BTG3 promoter and has antiproliferative effects through cell cycle arrest	(173)
**Peptide**	**Beta amiloid peptide**	Component of Alzheimer’s senile plaque	DNMT	Drecreases SAM/SAHlevels, promoting a global demethylation, through redox-dependent control over methionine synthase and methylation	Neuroblastoma cells	Soluble Aβ oligomers decreases intracellular glutathione levels by hampering cysteine uptake, followed by a global decrease in global DNA methylation	(174)
	**BCM7**	Natural Compound; food-derived peptide	DNMT	Decreases SAM/SAH levels, promoting decrease in cysteine levels, affecting redox status and methylation capacity of DNMTs	Neuroblastoma cells	Decreases cysteine absorption through opioid receptor activation. This reduction is followed by an increase of oxidized glutathione and an increase in DNA methylation	(175, 176)
	**GM7**	Natural Compound; food-derived peptide	DNMT		Neuroblastoma cells		
**Section 2. DNMT and HDAC Dual Inhibitors**							
**Polyphenol**	**Parthenolide**	Anti‐inflammatory (not approved)	HDAC1 and DNMT1	Inhibits DNMT1possibly through alkylation of the proximal thiolate of Cys1226 of the catalytic domain by its -methylene lactone	Colon Cancer cells	Inhibits HDAC activity by molecular docking, downregulates HIF-1alfa and inhibits NF-kB pathway	(177, 178)
					Melanoma cells	Reduces MITF-M transcript level and HDAC1 and protein level.	(179)
					Breast Cancer cells	Induces proteasomal degradation of HDAC1, thus increasing global histone acetylation and p21/p53 expression and induces cell death.	(179–181)
					Thyroid Cancer cells	Down-regulates DNMT1 expression possibly associated with its SubG1 cell-cycle arrest. Promotes global DNA hypomethylation and reactivates HIN-1 gene trough demethylation of its promoter	(181)
					Myeloid Leukemia (AML) cells	Inhibits DNMT1 and decreases gene expression of DNMT1 and BF-kB pathway. Induces cell cycle arrest and interrupts the binding of Sp1 to DNMT1 promoter, thus reactivates tumor suppressor genes and inhibites HIF-α	(178, 181)
	**Resveratrol (RVT/RSV)**	Natural Compound polyphenol (not approved);	HDAC1 and DNMT	Fits into the binding pocket of HDAC’s though interaction with amino acids of the catalytic site and interacts with the zinc ion, disrupting HDAC-zinc dependent activity.	Hepatoblastoma cells	Antiproliferative effect on all cell lines; showed specific inhibition of HDACs and in turn a histone hyperacetylation in HepG2 cells.	(182)
					Breast Cancer cells	Decreases PRMT5 EZH2 ATP2A3 and HDAC2 expression, increasing H3ac and H3K27 marks; increases global level of H3K9ac and H3K27ac marks through increasing KAT2A/3B expression. Reduces the enrichment of H4R3me2s and H3K27me3; and increases activating histone marks (H3K9/27ac) within the proximal promoter region of BRCA1, p53, and p21 restoring its expression.	(183, 184)
					Thyroid Cancer cells	Decreases DNMT1 activity and demethylates CpG sites at promoters regions in CRABP2.	(185)
	**EGCG**	Natural Compound; thiol anti-inflammatory	DNMT and HDAC	Inhibitor of DNMT nuclear activity by direct interaction with a hydrophilic pocket of DNMT1 and catalytic binding site	Squamous Cell carcinoma cells	Induces reversal hypermethytlation in RARβ, MGMT, p16INK4a, and hMLH1 promoter genes, promotes inhibition of cell growth.	(186, 187)
					Skin Cancer cells	Increases the levels of acetylation of histone H3 and histone H4 lysine residues through HDAC inhibition, leading to the upregulation of Cip1/p21 and p16INK4a.	(188)

Peptides are small proteins made up of fewer than 50 amino acids. Such compounds have several roles in the human body and can modulate epigenetic pathways, raising the exciting possibility of peptide-based therapy. Such peptides may be endogenous, or food derived. **Amyloid beta (Aβ),** the central component of Alzheimer’s senile plaque (AD), reduces global DNA methylation but increases DNA methylation in the Neprilysin gene promoting region, an Aβ-degrading enzyme (189). Soluble Aβ oligomers decrease intracellular glutathione levels by hampering cysteine uptake, followed by a global decrease in DNA methylation (174). **BCM7 and GM7** are food derived peptides produced by hydrolytic casein and gliadin digestion. They decrease cysteine absorption through opioid receptor activation in neuronal and gastrointestinal cells. This reduction is followed by an increase of oxidized glutathione and an increase in DNA methylation (175, 176) (**Table 4**, Section 1).

### Dual DNMT and HDAC Inhibitors

In most cancer types, altered DNMT and HDAC activity is observed (190). Therefore, some repurposed drugs that inhibit both DNMT and HDAC enzymes could improve efficacy over one-target agents (**Table 4**, Section 2).

**Berberine**, an isoquinoline alkaloid derived from *Berberis vulgaris* (191) and used to treat bacterial, parasitic, and fungal infections, has been repurposed as a DNMT and HDAC dual inhibitor (192). In multiple myeloma cell lines, berberine treatment showed downregulated DNMT1 and DNMT3A expression, restoring p53 expression through DNA hypomethylation (193). Berberine also inhibits Class I and II HDACs in lung cancer cell lines, down-regulates gene expression, and increases histone H3 and H4 acetylation (194). **EGCG** is a polyphenol found in green tea (*Camellia sinensis*) and is a known anti-inflammatory compound (195). It has recently been proposed as an inhibitor of DNMT by direct interaction with the catalytic site of DNMT (186–188). EGCG reduces cell growth and increases apoptosis in renal carcinoma cells through the upregulation of TFPI-2. In skin carcinoma cells, EGCG increases the levels of acetylation of histone H3 and histone H4 lysine residues through HDAC inhibition, leading to the upregulation of tumor-suppressor genes (188) (**Table 4**, Section 2). **Resveratrol** is a natural polyphenolic compound found in grapes and berries (196), and it has been proposed as a dual inhibitor of both DNMTs and HDACs. In breast cancer cell lines, resveratrol inhibits both HDAC and DNMT1 activity, decreases histone H3 lysine 27 methylation, and increases its acetylation (182–184). In thyroid cancer cell lines, treatment with resveratrol showed resensitization to therapy when in combination with retinoic acid through the demethylation of CpG sites at promoter regions of CRABP2 gene (185); the effect of resveratrol as a repurposed cancer drug was also investigated in clinical trials (NCT00256334, NCT01476592, NCT00433576). Finally, **parthenolide** is a terpenoid compound, isolated from *Tanacetum parthenium*, with anti‐inflammatory properties. Parthenolide downregulates HDAC1 gene expression (179) and increases histone acetylation (177, 180). It reverses drug resistance in some cancer cell lines (178) and restores silenced gene expression through a decrease in DNA methylation levels (181) (**Table 4**, Section 2).

### HDAC Inhibitors

As previously mentioned, the use of HDACi among the chemotherapeutic agents is growing (**Table 5**, HDACi). Hydroxamic and carboxylic acids are being studied as potential HDACi; for instance, drugs like **Vorinostat (SAHA)**, approved for psoriasis treatment, and **Valproic acid** (anticonvulsant) are currently included in several clinical trials against different types of cancers (236). A complete overview about clinical trials in some of the most studied HDACi repurposed, such as **Vorinostat**, **Valproate**, **Belinostat, Panobinostat,** and cyclic peptide **Romidepsin** is available (236) (**Table 5**, HDACi).

**Table 5:** Current HDACi repurposed drugs with applications in cancer therapy [*modified from Moreira-Silva et al. (9)].

Class	Compound	First indication	Epigenetic target	Drug-target interaction	Cancer model/New indication	Key features in mechanism	References
**HDAC inhibitors**							
**Acid**	**Valproate (VPA)**	Antiepileptic	HDAC class I and HDAC2	Inhibits HDAC class I activity by binding to the catalytic site and promotes proteasomal degradation of HDAC2.	Melanoma treatment	Potentiates karenitecin-induced apoptosis in multiple melanoma cell lines and on xenografted mice, however, fails to enhance chemotherapy effects on dacarbazine plus interferon-α-treated melanoma patients.	(197, 198)
					Colon Cancer cells	Reduces relative HDAC2 mRNA expression, preventing cell colony formation and migration.	(199)
					Non-small Cell Lung Cancer cells	Increases major histocompatibility complex (MHC) class I chain-related protein A (MICA) expression and sensitizes cancer cells to γδ T-cell-mediated killing.	(200)
					Colon Cancer Tumor cells	Synergistically reduces viability of cancer cells in combination with mytomicin C.	(201)
					Ovarian Cancer cells	Upregulates WWOX and P27 genes and interferes with the cell cycle by promoting apoptosis and inhibiting cell proliferation, both *in vitro* and *in vivo*.	(202)
**Phenolic**	**Artemisin**	Antimalarial	HDAC1, HDAC2 and HDAC6	Not described	Breast Cancer cells	Inhibits cell proliferation, cell migration, invasion and induces apoptosis. Also inhibits HDAC 1, 2, 6 and up-regulates BRCA1, 2/Ras/ERα/ERβ/PR/Her expression	(203)
	**Ginseng**	Nutraceutical (not approved)	HDAC	Not described	Lung Carcinoma cells	Inhibits HDAC activity, increases p21 expression and induces apoptosis.	(204)
	**HC toxin**	Natural Compound antiprotozoal (not approved);	HDAC	Not described	Breast Cancer cells	Inhibits cell proliferation and induces cell cycle arrest at G2/M and apoptosis in a dose-dependent manner.	(205)
					Neuroblastoma Cells	Induces cell cycle arrest and apoptosis, induces neuronal differentiation and inhibits invasive growth. Increases p-RB, p15, p16, p21, p27 expression, and reactivates the RB tumor suppressor pathway. Also induces H4 acetylation while inhibits HDAC activity	(206)
	**Psammaplin A (PsA)**	Enzimatic inhibitor Bromotyrosine Natural Compound (not approved);	HDAC III (SIRT1)	Inhibits HDAC activity via the coordination of zinc ion in catalytic pocket of HDAC with sulfhydryl group activated by a reducing agent.	Ovarian Cancer cells, Colon Cancer cells and Cervical Cancer cells	Displays significant cytotoxic activity, inhibits cell proliferation and upregulates expression of tumor-suppressor gene gelsolin in a dose-dependent manner	(207, 208)
					Endometrial Cancer cells	Inhibits cell proliferation, significantly induces H3 and H4 acetylation, upregulates expression of cyclin-dependent kinase inhibitor, p21, and downregulates expression of pRb, cyclins, and CDKs, promoting cell cycle arrest.	(209)
					Breast Cancer cells	Inhibits proliferation induces cell cycle arrest at G2/M and reduces SIRT1 activity protein expression levels and reduces nuclear SIRT1 levels. Increases p53 acetylation (target of SIRT1) and increases DRAM expression	(208, 210)
**Fatty acid**	**Sodium Butyrate**	Anti‐inflammatory	HDAC1	Not described	Gastric Cancer cells	Increases DAPK expression in human gastric cancer cells and this expression prompted apoptosis by decreasing FAK levels. Suggesting that DAPK expression prompts apoptosis by reducing the FAK protein level. Induce demethylation of the SFRP gene promoter	(211)
					Breast Cancer cells	Decreases cell proliferation induces cell cycle arrest at G1/G2 and decreases nuclear expression of DNA DSB repair proteins induced by etoposide (BRCA1 RAD51, ATM). Also increases H4 acetylation.	(212)
					Prostate Cancer cells	Inhibits HDAC1, 3 activity and induces H3, H4 acetylation, leading to hyperacetylation of H3 and H4 on the p21 promoter region, thus increasing p21 expression. Also induces cell cycle arrest, promoting apoptosis.	(213)
**Hydroxamic acid**	**Trichostatin A (TSA)**	Antifungal	HDAC class I, II and SIRT6	Hydroxamate. Pan-HDAC inhibitor, that obtain the binding energy associated with the strength of inhibition is derived from the bidentate chelation of hydroxamate	Breast Cancer cells	Decreases cell proliferation, inhibits HDAC activity, thus increases H4 hyperacetylation And increases ER acetylation and anti-tumor activity	(214)
					Myeloid Leukemia (AML) cells	Inhibits HDAC activity leds to histone hyperacetylation. Increases H4 acetylation and reduces Myc expression ZNF278 (Myc’s coactivator), NM1, HOXB6 and MKRN3	(215)
					Esophageal Squamous Carcinoma cells	Decreases cell proliferation, induces cell cycle arrest at G1, down-regulates cell growth by inhibiting the activation of the PI3K/Akt and ERK1/2 pathways, and increases H4 acetylation levels	(216)
					Prostate Cancer cells	Increases apoptosis induces p21 expression and represses TMPRSS2-ERG expression AND affect acetylation status of p53 by inhibiting HDAC activity. Disrupts the epidermal growth factor receptor (EGFR),-STAT3 pathway, thus, inhibits proliferation in CRPC cells. Increases H4K16acetylation and promotes gene transcription, moreover decreases phospho-Akt pathway	(217, 218)
					Pancreatic Cancer cells	Restores cellular differentiation, reduces proliferation and restores p21 expression. Increases NDGR1 mRNA expression, also increases hypoxic responses	(219)
					Colon Cancer cells	Decreases cell growth and promotes apoptosis, down-regulates DNMT1 and HDAC1 expression, increasing p21, p27 and p57 expression	(220)
					Hepatocellular Carcinoma cells	Increases H3K9 and H3K27 acetylation and increases SERCA3 mRNA expression levels and promote ATP2A3 gene expression	(221)
	**Vorinostat (SAHA)**	Psoriasis disease treatmenr	HDAC class I, II and IV	inhibits HDAC activity by binding to the pocket of the catalytic site processes by removing acetyl groups from proteins	Advanced Prostate Cancer treatment	In a phase II trial, it was associated with significant toxicities limiting efficacy assessment in patients with disease progression on one prior chemotherapy	(222, 223)
					Follicular and Mantle Cell Lymphoma treatment	In a phase I trial in follicular and mantle cell lymphomaoral vorinostat was well tolerated up to 200mg bd for 14 consecutive days every 3 weeks in Japanese patients with NHL. Shown favorable results	(224)
	**Panobinostat**	HDACi multiple myeloma	HDAC	Pan-HDAC inhibitor, blocks the enzymatic activity of HDAC	Multiple myeloma treatment	It has improved progression-free survival when combined with bortezomib and dexamethasone in patients with relapsed multiple myeloma who previously received bortezomib and an immunomodulatory agent	(225)
**Depsipeptide**	**Burkholdacs A**	Pathogen bacteria (not approved)	HDAC1, HDAC6	Inhibits HDAC catalytic activity by reduction of the disulfide bond which generates a free thiol group that interacts with the catalytic site in HDACs.	Brain Cancer cells	In at least six cancer cell lines, it has shown superior HDACi activity over Ramidopsine (approved HDACi). Burkholdacs A presents more affinity for HDAC1 and was determined to be superior than B with respect to its HDAC1 inhibitory activity and isoform selectivity toward HDAC1 over HDAC6 and antiproliferative activity.	(226)
					Colon Cancer cells		
					Lung Cancer cells		
					Ovary Cancer cells		
					Stomach Cancer cells		
					Prostate Cancer cells		
	**Thailandepsin (Burkholdacs B)**	Pathogen bacteria (not approved)	HDAC6		Cervical Cancer cells		(226, 227)
					Breast Cancer cells		(227)
	**Spiruchostatin A**	Pathogen bacteria (not approved)	HDAC1 and HDAC6	Structural similarity with HDAC inhibitor FK228 (Romidepsin) which interacts with the active-site zinc in its reduced form, preventing it from interacting with substrate.	Breast Cancer cells	Increases acetylation levels of specific lysine residues of histones H3 and H4.	(228, 229)
					Ovarian Cancer cells		
					Brain Cancer cells		
					Colon Cancer cells		
	**Apicidin**	Antiprotozoal for Malaria	HDAC3, HDAC4 and HDAC8	Cyclic tripeptide that chelate the active site zinc ion through the terminal carbonyl, hydroxy and/or amino functional groups	Promyelocytic Leukemia treatment	Inhibits cell proliferation an cycle arrest, promoting cell death. Increases H4 acetylation and inhibits HDAC activity, thus increases p21 expression.	(230)
					Lung, Colon and Pancreatic Cancer cells	Induces DNA demethylation via HMT suppression, reduces HP1 and DNMT1 recruitment to genes’ promoter and induces p16, SALL3, and GATA4 expression. Also, decreases SUV39 and G9a expression in lung cancer cell lines.	(231)
					Cervical Cancer cells	Induces demethylation of CpG islands of the 1st exon of the PDH2 gene AND induces PHD2 and p21 gene expression and inhibits cell proliferation.	(232)
					Breast Cancer cells	Increases H3 and H4 acetylation and reduces ERalfa and ERb expression. Increases p21 and p27 expression and reduces cyclin D1 and cyclin E expression. Also reduces cell proliferation, thus promotig apoptosis.	(233)
					Endometrial Cancer cells	Increases H3 acetylation and reduces HDAC3, 4 expression, decreases cell proliferation and induces apoptosis.	(217)
					Ovarian Cancer cells	Decreases HDAC activity, reduces HDAC4 expression and blocks cell migration and invasion. Increases H3 and H4 acetylation and increases RECK expression through reducting the binding of HDAC4 to the Sp1 of its promoter, while reduces MMP-2 expression.	(234)
					Oral Squamous Cell Carcinoma cells	Inhibits cell growth, proliferation and reduces HDAC8 expression. Induces apoptosis and autophagy AND increases H4 acetylation.	(235)
	**Platycodi**	Nutraceutical (not approved)	HDAC	Not described	Lung Carcinoma cells	Inhibits HDAC enzymatic activity and induces the expression of p21. Stimulates cell death and inhibits cell proliferation.	(204)

Compounds with HDACi potential have been found in plants. **Ginseng** (*Panax ginseng*) is a popular plant extract commonly used in South Korea and traditional Chinese medicine, which contains several compounds (ginsenosides) with pharmacological properties (144). **Platycodi radix** (*Platycodon grandiflorum*), commonly known as balloon flower, is used to treat many diseases related to obesity in East Asia (237). Recently, Byun and cols. demonstrated that ginseng and platycodi have significant HDACi activity in Lung Carcinoma cell lines, thus upregulating p21 gene expression and promoting cell death (204). **HC toxin** is a cyclic tetrapeptide derived from a plant-fungal parasitic-association between *Helminthosporium carbonum* (ascomycetes) and its host, (commonly *Poaceae* plants family). It was reported as a Maize Histone Deacetylase inhibitor (238) and proposed as an analog of **Apicidin** and **Artemisin**, a fungal metabolite (239), and antimalarial drug, respectively; with antiprotozoal HDACi activity proved for Malaria (*Plasmodium berghei*) in mice. However, recently HC-toxin has been rediscovered and identified as HDACi in different cancer cell lines (205). In breast cancer and neuroblastoma cell lines, HC toxin inhibited HDAC activity and promoted cell proliferation inhibition, cellular death, and induced H4 acetylation (205, 206). **Artemisin** has been repurposed as an HDAC1, HDAC2, and HDAC6 inhibitor in the breast cancer cell line MCF‐7 (203) (**Table 5**, HDACi).

**Psammaplin A (PsA)** is a phenolic compound that derives from the marine sponge-association, *Poecillastra sp.* and *Jaspis sp.*, *(Pseudoceratina purpurea)* whose active substances are monomers of thiol groups with enzymatic inhibition activity (210, 240). These monomers play a key role for both HDACi and DNMTi activity (241). In endometrial cancer cells, PsA showed HDAC1 and HDAC6 inhibition, reduction of HDAC1 expression the elevation of histone H3 and H4 acetylation, induction of cell cycle arrest, and apoptosis (208, 209). **Burkholdacs A** **and B**, with a structure similar to **Thailandepsin A**, was identified as a novel HDACi through the systematic overexpression of transcription factors associated with *Burkholderia thilandensis* (227). They are bicyclic depsipeptide compounds, proposed as potent HDACi in brain cancer cells, but also in other cancer cell lines (226). Using a panel of 39 human cancer cell lines, burkholdacs have shown superior HDACi activity over Ramidopsine (approved HDACi) in at least six cancer cell lines (226). Burkholdacs’ affinity for HDAC1 is greater than that for HDAC6. Structural changes in burkholdacs A and B structures may increase their activity and selectivity, giving rise to isoform selective inhibition of HDACs therapeutical potential (226) (**Table 5**, HDACi). Other depsipeptides have also been studied for repurposing. **Spiruchostatin A, and Plitidepsin (Aplidin)** are natural depsipeptides derived from *Pseudomonas sp.* (228) and *Aplidium albicans* (242), respectively. In cancer cell lines, reduced spiruchostatin A effectively inhibited HDAC1, an effect not observed when oxidized, and it showed an increase in the acetylation levels of specific lysine residues of histones H3 and H4 (228). Plitidepsin is currently in clinical trials to treat multiple myeloma (243, 244) but it has also displayed interesting properties against hematological malignancies (245). Some depsipeptides display a greater affinity for HDAC1 than HDAC6 and class II HDACs, but this does not appear to limit their activity as anti-cancer agents judging by *in vitro* effects in cancer cells (208, 226, 228). Structure-function studies on depsipeptides can lead to the generation of chemical analogs with enhanced selectivity as HDACi drugs (**Table 5**, HDACi).

### HAT, HMT, HDM, and BET Inhibitors

Recently, HATi, HMTi, HDMi, and BETi have become of great interest for personalized cancer treatment. Multiple studies have consistently shown the enormous potential of known drugs and compounds for DR as epigenetic modulators (**Table 6**, HATi, HMTi, HDMi, and BETi).

**Table 6:** Current HAT HMT, HDM, and BET inhibitors repurposed with epigenetic applications in cancer therapy [*modified from Moreira-Silva et al., (9)].

Class	Compound	First indication	Epigenetic target	Drug-target interaction	Cancer model/New indication	Key features in mechanism	References
**HAT, HMT, HDM, and BET inhibitors**							
**HATi**	**Anacardic acid**	Anti‐inflammatory; food-derived (not approved)	HAT/Ep300 and Tip60	Not described	Cervical Tumor cells	Inhibits Tip60 HAT and ATM acetylation. Promotes resensitizing tumor cells to the cytotoxic effect of radiation.	(246)
					Myeloid Leukemia cells	Inhibits p300 HAT activity. Also, inhibits NF-kB activation, inhibits IkBalfa activation, p65 acetylation and nuclear translocation. It potentiates apoptosis via TNF-induced caspase activation and suppresses the expression of genes involved in invasion and angiogenesis.	(247, 248)
					T‐Cell Lymphoma cells		
					Lung Cancer cells		
					Prostate Cancer cells		
	**Garcinol**	Antioxidant benzophenone (not approved);	HAT2B/Ep300	Not described	Cervical Cancer cells	Inhibits p300 and KAT2B activity, HAT activity and induces apoptosis.	(249)
					Breast Cancer cells	Decreases H3K18 acetylation and increases DNA damage signaling markers. Inhibits HAT activity and induces cell proliferation arrest.	(250)
					Hepatocellular Carcinoma cells	Decreases HAT activity and inhibits STAT3 activation through acetylation. Decreases proliferation, tumor growth, survival and angiogenesis.	(251)
					Esophageal Carcinoma cells	Decreases p300/CBP levels, induces cell cycle arrest, thus induces apoptosis and inhibits migration and cell invasion and proliferation. Inhibits metastasis and inhibit HAT and its cofactors, decreasing TGF-beta pathway.	(252)
	**Plumbagin**	Nutraceutical quinone (not approved);	HAT3B/p300	Inhibits p300 HAT activity (non-competitive), through a single hydroxyl group of plumbagin that makes a hydrogen bond with the lysine 1358 residue of the p300 HAT domain.	Liver Carcinoma cells	Inhibits p300 HAT activity AND inhibits p300-mediated acetylation of p53 AND reduces H3 and H4 acetylation AND induces apoptosis AND modulates the enzymatic activity of p300. *in vivo*: reduces H3 acetylation.	(253)
	**Lunasin**	Natural Compound; food-derived peptide	HAT	Not described. Possibly a competitive inhibitor	Cancer preventive in mouse Fibroblasts	Suppresses foci formation in mice fibroblast cells induced by chemical carcinogens by the RGD motif and its chromatin-binding property, binding to deacetylated histones, and the reduction of histone acetylation.	(254)
**HMTi**	**Allantodapsone**	Antibiotic (Dapsone-derivated)	H4R3me	Inhibitory activity toward PRMT1	Hepatocellular Carcinoma cells	Inhibits cellular H4R3 methylation to the same level as AMI-1, while the H3K4 methylation level is barely impacted.	(255)
	**Ribavirin**	RSV infections and Hepatitis C	EZH2	Not described. Possibly a selective inhibitor of EZH2	Solid Tumors (Atypical teratoid/rhabdoid tumor)	Inhibits cell growth, induces cell cycle arrest and apoptosis. Also inhibits eIF4E and EZH2 activity decreasing its expression levels. Impairs cell migration, invasion and adhesion. In osteosarcoma enhances chemosensitivity.	(230, 256)
					Breast, Brain, Cervical, Colon and Prostate Cancer cells	Decreases EZH2 expression, inhibits HMT activity and decreases H3K27me3. Induces variable growth inhibition and downregulation of EZH2, eIF4E and IMPDH1.	(257)
	**Hydroxychloroquine (HCQ)**	Antimalarial/Arthritis	PRC2	Disruption of PRC2-EED complex by allosteric PRC2-EED binding inhibition within the H3K27me3-binding pocket, thus antagonizing the PRC2 catalytic activity	Multiple Myeloma Cells	Decreases H3K27me3 levels in MM cells 3 by disrupting the H3K27me3- EED interaction within the PRC2 complex. Suggesting that its anti-tumor activity might rely on the reactivation of genes abnormally silenced via H3K27 hypermethylation.	(258)
**HDMi**	**Clorgyline**	MAO inhibitor	LSD1	Not described	Bladder Cancer cells	Induces DNA demethylation, inhibits LSD1, decreasing H3K4me2 and H3K4me, establishes an active chromatin state. Inhibits cell growth induces the expression of previously silenced genes by enriching H3K4me2 and H3K4me1 histone marks.	(259)
					Colon Cancer cells		
					Promyelocytic Leukemia Cells		
	**Geranylgeranoic acid**	Natural Compound (not approved)	LSD1	Not described	Neuroblastoma cells	Inhibits LSD1 activity, induces NTRK2 gene expression and increases H3K4me2. Moreover decreases cell proliferation.	(260)
	**Pargyline**	MAO‐B inhibitor; antihypertensive	LSD1	Not described	Prostate Cancer cells	Inhibits cell migration and invasion AND inhibit EMT AND induces E-cadherin expression AND inhibits N-cadherin and Vimentin expression AND delayed PCa transition to CRPC AND decreases PSA expression AND decreases H3K4 and H3K9 di-methylation.	(261)
	**Tranylcypromine**	Severe depression	LSD1	Not described	Glioblastoma cells	Induces cell death AND inhibits LSD1 activity AND increases cell sensitivity to HDACi.	(262, 263)
	**Polymyxin A/B**	Antibiotic	LSD1	Inhibits LSD1 by competition with its substrate at the enzyme’s cleft entry	Chemical inhibition of LSD1 assay	*In vitro* assays demonstrated that quinazoline core can represent a privileged scaffold for developing inhibitors that target epigenetic enzymes.	(264)
**BETi**	**Azelastine**	Anti-histaminic	BET-BRD4	Inhibits BRD4 through interactions with several key residues of the acetyl lysine binding pocket	Structural *in silico* assays by docking-based method	Docking-based database screening identified Azelastine drug as a promising novel template exhibiting binding affinity better than the control lead (+)-JQ1 for the human BRD4. Azelastine is having a low molecular weight, which gives a scope of further chemical modification to enrich its binding affinity for BRD4.	(265)
	**Nitroxoline**	Antibiotic	BET-BRD4	Occupies the acetylated lysine binding pocket of the first bromodomain of BRD4	MLL Leukemia cells	Prevents the binding of BRD4 to acetylated H4	(266)

#### HAT Inhibitors

**Anacardic acid**, a small molecule obtained from cashew nutshell liquid with known antitumor activity, inhibits the p300’s and PCAF’s HAT activity. Anacardic Acid is not specific to any particular HAT group, but it can be used to synthesize other specific HAT activity modulators based on this molecule (246). **Plumbagin** is an *in vivo*, potent acetyltransferase inhibitor, hydroxynaphthoquinone isolated from the roots of *Plumbago Rosea*. A single hydroxyl group in Plumbagin confers its HATi properties. Replacing this group with other chemical moieties results in complete loss of its inhibitory activity. Plumbagin has also been reported to suppress the activation of NFκ-B, leading to apoptosis potentiation. Plumbagin may be a potential anticancer agent, but its cell toxicity properties could be the main limitation of its use as a therapeutic molecule (253). **Garcinol** is a potent inhibitor of the p300 and PCAF HATs. It inhibits *in vivo* histone acetylation in HeLa cells but does not affect histone deacetylation. Garcinol suppresses chromatin transcription dependent on HAT p300 but does not affected transcription of DNA (249). **Lunasin** is a 43 amino acid peptide found in soybean, barley, wheat, and rye. Previous studies have shown that lunasin can suppress the proliferation and migration of cancer cells with no effect on wild-type cells. Lunasin is a competitive inhibitor of HATs. It inhibits histone acetylation and regulates the cell cycle. This binding is probably achieved through its helical structure, similar to chromatin-binding protein structures (267) (**Table 6**, HATi).

#### HMT Inhibitors

**Allantodapsone** was recovered from a virtual screening based on the PRMT1 structure. Allantodapsone inhibits H4R3 methylation in the hepatocellular carcinoma cell line HepG2 while leaving H3K4 methylation unaffected (255). **Ribavirin** is an antiviral drug that has become of interest as a therapeutic agent in cancer. Ribavirine selectively inhibits pediatric osteosarcoma and improves chemosensitivity (256). It also possesses *in vitro* growth inhibitory effects against various malignant cell lines at clinically reasonable concentrations; also, ribavirin treatment results in the reduction of EZH2 at RNA and protein levels, inhibition of EZH2 enzyme activity, and reduction of H3K27 methylation (257). The anti-malarial drug, **hydroxychloroquine**, has also been effective in treating rheumatoid lupus, arthritis, and *porphyria cutanea tarda*. Structural experiments have shown that hydroxychloroquine inhibits the allosteric binding of PRC2 to EED within the H3K27me3-binding region, thereby antagonizing the catalytic function of the PRC2. These findings suggest a new epigenetic function of hydroxychloroquine with possible therapeutic repositioning (258) (**Table 6**, HMTi).

#### HDM Inhibitors

**Clorgyline** is a selective MAO A inhibitor- used as an antidepressant until severe dietary adverse effects are commonly known as the “cheese effect” were reported for this drug (268). As a member of MAO inhibitors, clorgyline can also inhibit LSD1, and it has been demonstrated to have cell-type dependent synergic effects when combined with DNMTi (259). **Geranylgeranoic acid**, an acyclic diterpenoid present in medicinal plants, has recently been found to be a potent inhibitor of recombinant LSD1. Geranylgeranoic acid inhibits the proliferation and induces a neuronal phenotype through increasing the abundance of H3K4me2 of NTRK2 gene promoter in human SH-SY5Y-derived neuroblastoma cells (260). **Pargyline**, a MAO B selective inhibitor with antidepressant activity, affects the transition from androgen-dependent to androgen-independent in prostate cancer. Inhibition of LSD1 with a concomitant reduction of H3K4me2 and H3K9me2 levels have been reported for pargyline. Pargyline, in combination with androgen deprivation therapy, could be an effective adjunctive treatment for advanced prostate cancer (261). Unlike selective MAO inhibitors such as pargyline, non-selective MAO inhibitors strongly repress the nucleosomal demethylation of histone H3K4. **Tranylcypromine**, a drug used in treating severe depression, has demonstrated strong LSD1 inhibitory effects with an IC50 of less than 2 mM (262). Tranylcypromine contributes to GBM cell synergistic apoptosis in association with other HDAC inhibitors (263). Recently, molecular docking studies have highlighted the potential of approved drugs such as **decitabine**, **entecavir**, **abacavir**, **penciclovir,** and **DZNep** as KDM5B inhibitors. Their role as HDMi could be of great importance in lung cancer, melanoma, hepatocellular carcinoma, gastric cancer, and prostate cancer, among others. Decitabine is a DNMTi used in myelodysplastic syndrome (MDS), abacavir, entecavir, and penciclovir are antivirals used in the treatment of HIV, hepatitis B, and herpes infections, respectively. DZNep is a specific HMTi with promising results in cancer immunotherapy (269). Finally, **Polymyxin B and polymyxin E** are antibiotics used in multidrug resistant bacterial infections. These compounds were shown to inhibit LSD1 by competition with its substrate at the enzyme’s cleft entry. Polymyxins have significant side effects that limit their application to untreated infections, but they could still be the target of drug repurposing for other diseases, such as leukemia (264) (**Table 6**, HDMi).

#### BET Inhibitors

**Azelastine**, a selective H1 antagonist, was found to be a promising BETi, displaying a stronger binding affinity than BETi control JQ1 for human BRD4 by docking-based methodologies. These findings highlight the importance of computational methods for molecular drug design and will uncover new BRD4 inhibition candidates (265). The antibiotic approved by FDA, **nitroxoline**, disrupts the association of BRD4 bromodomain with acetylated H4. Nitroxoline has shown strong selectivity at inhibiting all BET family members compared with non-BET proteins. By causing cell cycle arrest and apoptosis, nitroxoline successfully prevents the proliferation of MLL leukemia cells. The possible use of nitroxoline and its derivatives as BET inhibitors in BET related diseases is now under investigation (266) (**Table 6**, BETi).

## Concluding Remarks

Drug repositioning has emerged as a viable strategy to increase drug discovery’s overall productivity, resulting in a new and cheaper way to generate alternative therapies for various diseases, including cancer. The drug repositioning approach is growing due to a broad range of reposition candidate molecules that already have clinical and toxicity profiling developments. One factor that has strongly driven this approach is the increasing availability of biomedical data, including genomic data, which covers various aspects of cellular mechanisms, opening a search that is not restricted to biological factors involved in a disease. This omic perspective allows the deduction of complex interactions that can be inhibited or treated to cure or reverse a pathological condition. Advances in complementary bioinformatic analytical methods provide critical substrate candidates that enable their systematic evaluation. Therefore, a window of opportunity opens where the reuse of previously synthesized drugs can be investigated and given a new direction. Epi-DR has already shown a profit in epigenetics and cancer treatment, where it has proven its efficacy. Indeed, many epidrugs emerged this way, such as 5-azacytidine and 5-aza-2′-deoxycytidine (decitabine) (146), Hydralazine (156), Vorinostat (SAHA), and Valproic acid (236).

Epigenetic alterations are considered to be among the earliest and most comprehensive genomic aberrations occurring during carcinogenesis, and therefore it has been classified as a hallmark of cancer (270). The impact of epigenetics in understanding cancer has been of great interest in recent years, and even more due to the advancement of the genomic era. Several works demonstrate the importance of epigenetic biomarkers that can predict the response or prognosis in various types of cancer. The promoter methylation of the MGMT gene in gliomas is a clear example, where it helps to indicate the use of precision medicine through the drug temozolomide (271). Another example is found in EHZ2 enzyme alterations, which indicate a poor prognosis in breast, prostate, and other types of cancers.

Epigenetic mechanisms have great flexibility to respond to environmental changes and modify gene expression. Consequently, search for artificial ways to induce epigenetic remodeling, which could improve therapy in the event of a disease as cancer. Therefore, the implementation of epigenetic therapies opens a new panorama for the fight against cancer. Epidrugs show enormous potential for clinical use, especially in cancer, because in these diseases, an epigenetic imbalance is a well-known characteristic that is both of origin, development, and severity of tumors.

Even though there are already some epidrugs approved by the FDA and the current knowledge about various mechanisms involved in gene regulation, promoted by the advancement of technologies that expand the information on specific epigenetic mechanisms, challenges remain in identifying epigenetic modifications of cancer and targeting them for therapeutic purposes. Among them stands out that epigenetic changes can be diverse in the types of cancer and between the different clinical phases and those that are dependent on environmental conditions. Therefore, we must distinguish between the dysregulation of driver genes and those whose changes are secondary to these. Also, the generation of epigenetic therapies as well as the molecular mechanisms that coordinate them is subject to understanding, and much research is still required of several of them to safely transport them to the clinic. However, identifying epigenetic alterations that affect the tumor’s fate and behavior finding drugs that target them are some of the promises of epigenetic therapy in cancer.

In this sense, the concept of reusing a medicine offers a broad scope to investigate the hidden potential behind the medicine and to recycle it. The reincorporation of a drug with the potential to remodel epigenetic characteristics, which are beneficial for cancer management, is of great interest to the field. Offering great advantages in drug development times could lead to precision medicine therapy with new and clearly encouraging prospects for the future (**Figure 4**).

## Author Contributions

MM-C and MM-R wrote and designed the manuscript. RG-B coordinated, wrote, and designed the manuscript. RG-B and VJ-G revised the manuscript. VJ-G elaborated on the figures. CA-C wrote and revised the manuscript, and LH coordinated and directed the review development. All authors contributed to the article and approved the submitted version.

## Funding

This work was supported by the Consejo Nacional de Ciencia y Tecnología (CONACyT) by the Fondo Sectorial de Investigación en Salud y Seguridad Social (FOSISS, grant no. SALUD-2017-2-290041). Marco Antonio Meraz-Rodriguez is a masters student in the “Programa de Maestría y Doctorado en Ciencias Bioquímicas, UNAM”, and received a fellowship from CONACyT (CVU 659273, no. 481908).

## Conflict of Interest

The authors declare that the research was conducted in the absence of any commercial or financial relationships that could be construed as a potential conflict of interest.
